# Interaction of Glycolipids with the Macrophage Surface Receptor Mincle – a Systematic Molecular Dynamics Study

**DOI:** 10.1038/s41598-018-23624-8

**Published:** 2018-03-29

**Authors:** Christian A. Söldner, Anselm H. C. Horn, Heinrich Sticht

**Affiliations:** Bioinformatik, Institut für Biochemie, Emil-Fischer-Centrum, Friedrich-Alexander-Universität Erlangen-Nürnberg, (FAU), Fahrstraße 17, 91054 Erlangen, Germany

## Abstract

Synthetic analogues of mycobacterial trehalose-dimycolate such as trehalose acyl esters have been proposed as novel adjuvants for vaccination. They induce an immune response by binding to the macrophage C-type lectin receptor Mincle. The binding site of trehalose is known, but there is yet only very limited structural information about the binding mode of the acyl esters. Here, we performed a systematic molecular dynamics study of trehalose mono-and diesters with different chain lengths. All acyl chains investigated exhibited a high flexibility and interacted almost exclusively with a hydrophobic groove on Mincle. Despite the limited length of this hydrophobic groove, the distal parts of the longer monoesters can still form additional interactions with this surface region due to their conformational flexibility. In diesters, a certain length of the second acyl chain is required to contact the hydrophobic groove. However, a stable concomitant accommodation of both acyl chains in the groove is hampered by the conformational rigidity of Mincle. Instead, multiple dynamic interaction modes are observed, in which the second acyl chain contributes to binding. This detailed structural information is considered helpful for the future design of more affine ligands that may foster the development of novel adjuvants.

## Introduction

The development of novel adjuvants, i. e. substances enhancing the cellular immune response upon vaccination, is a main objective in the research against infectious diseases such as tuberculosis, which leads to about two million deaths per year^1^. Recently, the macrophage surface receptor monocyte inducible C-type lectin CLEC4E (Mincle) has been identified as a key molecule conveying a potent immune reaction to glycolipids of the mycobacterial cell wall such as trehalose-6-6′-dimycolate (TDM), also known as cord factor^2,3^. The signal transduction of Mincle relies on an associated adapter protein Fc receptor *γ* (FcR *γ*) chain containing an immunoreceptor tyrosine-based activation motif. An activation of the Card9-Bcl10-Malt1 complex occurs via the kinase Syk and induces the expression of chemokines, growth factors (e. g. G-CSF) and cytokines (e. g. IL-6, TNF)^4^. This leads to a further recruitment of inflammatory cells and the consecutive development of granulomas which encapsulate the sites of infection preventing thereby the dispersion of mycobacteria^5^.

**Figure 1:**
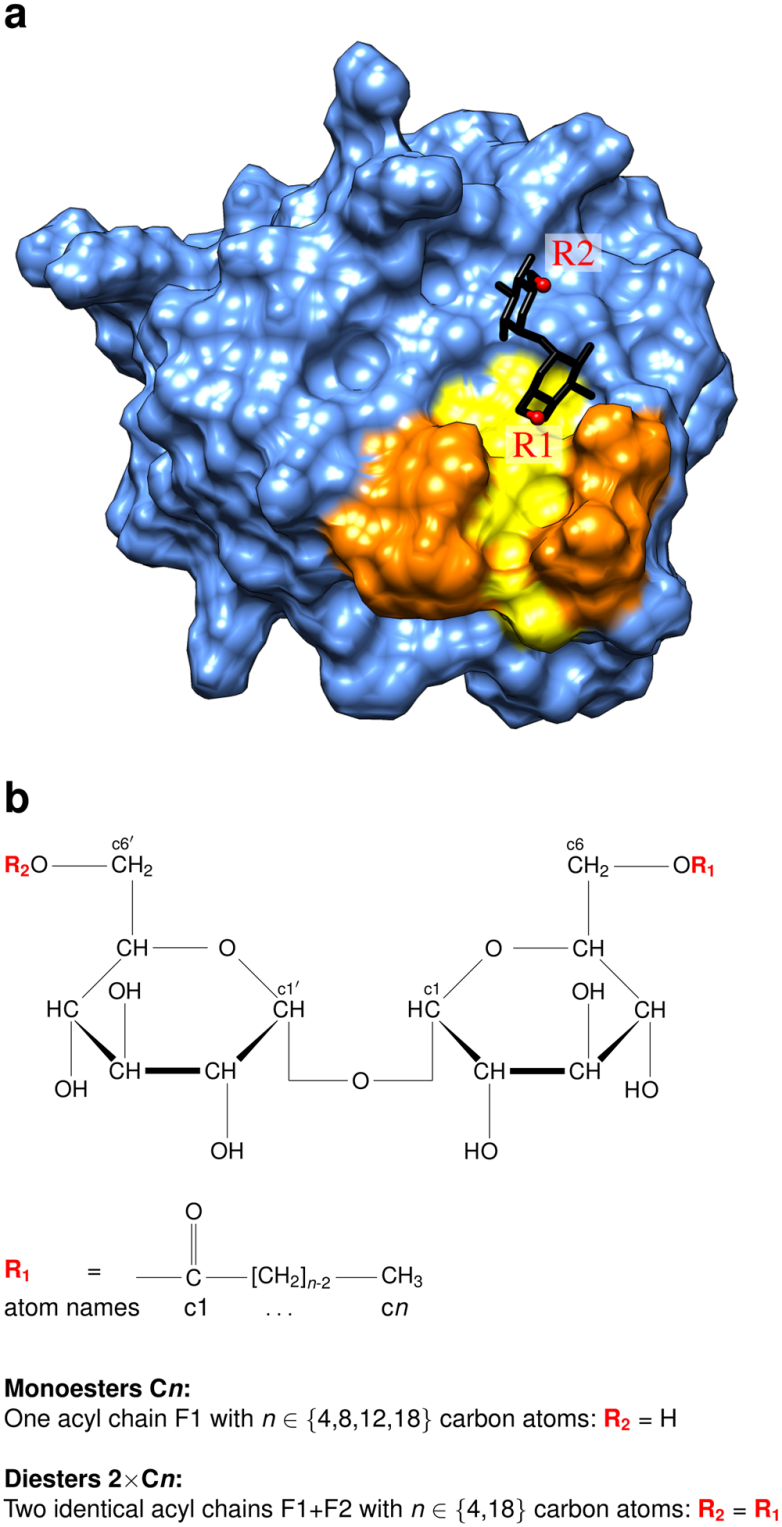
Structure of Mincle and the glycolipid ligands. (**a**) Structure of the Mincle carbohydrate recognition domain (PDB code: 4ZRV; ref.^7^). The protein domain is shown in surface presentation and the hydrophobic groove representing a putative ligand binding site is highlighted in yellow. Residues L172, V173, F197, and F198 that line the hydrophobic groove are marked in orange. The trehalose moiety is shown as stick presentation and the attachment sites of the first and second acyl chain are labelled R1 and R2, respectively. (**b**) Structure of trehalose acyl esters. Trehalose is a disaccharide consisting of two glucose moieties. Atoms are referred to by lower case characters in the present study. Monoesters are esterified at the c6 atom, diesters both at the c6 atom of the first and the c6′ atom of the second glucose moiety.

Synthetic trehalose acyl esters with one or two saturated alkyl chains, which represent less toxic TDM analogs, have been shown to bind to Mincle^6–9^ and to induce a similar immune reaction as TDM^8,10,11^. The interaction of synthetic trehalose acyl esters and Mincle has been investigated by X-ray crystallography^6,7^ revealing that the trehalose moiety binds close to a hydrophobic groove of the Mincle carbohydrate recognition domain (CRD) (Fig. 1a). Site directed mutagenesis suggests that residues on the surface of this groove form the binding site for the fatty acid moieties of the trehalose acyl esters^6^. However, a detailed structural characterization of the interaction between Mincle and these acyl chains proved to be difficult: In an X-ray structure of Mincle in complex with trehalose monobutyrate, only the first two carbon atoms of the fatty acid could be modeled from the electron density map suggesting that the remainder might be flexible^7^. There is another observation that is yet poorly understood on a structural level: Despite the limited length of the hydrophobic groove, which allows to accommodate roughly six carbon atoms^6^, trehalose esters with longer fatty acids were shown to bind Mincle with higher affinity and cause stronger activation^7,8^. Although long monoesters are already significantly active^10^, diesters with the same length of the individual acyl chains lead to a further enhanced immune reaction^8^. It has been proposed in this context that one acyl chain of the diesters might reside within the hydrophobic groove whereas the other one could have a different binding site^12^. One method that allows to elucidate structural details of such flexible interactions, which can hardly be assessed by crystallographic analysis, are molecular dynamics (MD) simulations. This method has been previously applied to investigate the binding of glucose monoesters to Mincle^11^, whereas there is yet no correspondent information available for trehalose mono- and diesters. Therefore, we used MD simulations to characterize how trehalose esters interact with Mincle. In order to get insight into (i) the influence of the length of the acyl chain upon binding affinity and (ii) the difference between monoesters and diesters, we performed a series of all-atom molecular dynamics simulations of Mincle bound to different trehalose ester ligands in explicit water. We examined trehalose-6-monoesters with acyl chains comprising 4, 8, 12, and 18 carbon atoms. Moreover, trehalose-6-6′-diesters with two identical chains of either 4 or 18 carbon atoms were studied. An overview of the simulated ligands is shown in Fig. 1b.

## Methods

### Preparation of Starting Structures

The starting structures were derived from a high-resolution crystal structure of trehalose monobutyrate bound to bovine Mincle (PDB code 4ZRV)^7^. In this structure, the binding site of the sugar is characterized by numerous specific protein-sugar interactions including a bridging calcium ion. For that reason, we adopted this sugar binding site in our simulations and focused on the conformational properties of the lipid moieties. From the three copies of Mincle in the PDB entry 4ZRV, we selected chain C because in this copy the first two butyrate carbon atoms were resolved in addition to the trehalose moiety. Crystal water and Ca^2+^ ions bound to this chain were kept. We constructed structures for the different fatty acids using Avogadro 1.1^13^ and converted them to Amber prep files with antechamber. They were assigned Glycam06j-1^14^ atom types. Charges were determined by performing a RESP/ESP fit with the RESP/ESP Charge Derive Server^15^ using Gaussian09^16^ RESP-C2 (HF/6-31G*//HF/6-31G*) (for details and Amber Prep file see Supplement). Taking the first two carbon atoms as anchor points, the missing coordinates of the various acyl chains were added with leap resulting in a starting structure in which the acyl chain was inserted into the hydrophobic groove. To avoid a bias resulting from the conformation of the starting structure, an alternative starting conformation was generated for a second simulation run, in which the fatty acid was oriented towards the solvent. This was done by changing the torsion angle between the trehalose and the fatty acid. In the case of the diesters, a second acyl chain was added with UCSF Chimera^17^ in an extended conformation oriented towards the solvent. The trehalose acyl monoesters and diesters in our MD simulations were assigned atom types and force field parameters of the Glycam force field. This step also included generation of a modified sugar type to allow the attachment of a second acyl chain to trehalose (for details and Amber Prep file see Supplement). A single Cl^−^ was added to every starting structure for electrical neutralization. The molecules were solvated in a capped octahedral periodic water box with at least 12 Å distance from the borders to the solute. Table 1 shows an overview of the systems investigated.

**Table 1:** Overview of the investigated systems.

System	Run	Acyl chains		Initial position	Atoms	Water molecules
		Number	Length			
C4	1	1	4	inside groove	23, 385	7, 083
	2			outside groove	19, 230	5, 698
C8	1	1	8	inside groove	23, 802	7, 218
	2			outside groove	19, 563	5, 805
C12	1	1	12	inside groove	25, 275	7, 705
	2			outside groove	22, 596	6, 812
C18	1	1	18	inside groove	32, 136	9, 986
	2			outside groove	30, 168	9, 330
2 × C4	1	2	4	F1 inside, F2 outside	23, 756	7, 203
	2			F1 + F2 outside groove	23, 879	7, 244
2 × C18	1	2	18	F1 inside, F2 outside	32, 300	10, 023
	2			F1 + F2 outside groove	40, 115	12, 628

The ligands were composed of trehalose esterified to one (via c6 atom) or two acyl chains (via c6, c6′ atom) as shown in Fig. 1a. The acyl chains are named C4 (butyrate), C8 (octanoate), C12 (dodecanoate), C18 (octadecanoate) according to the number of carbon atoms. In diesters, the two acyl chains are referred to as F1 and F2.

### Molecular Dynamics Simulations

Molecular dynamics simulations were performed using version 14 of the Amber molecular dynamics software package (ambermd.org)^18^. Glycam parameters^14^ were used for both sugar and acyl chains similar to a previous study of related glycolipids^11^. The protein was simulated with the *ff14SB* force field^19^. Ca^2+^ ions were described using the HFE/IOD 12-6 parameters by Li and Merz^20^. The simulations followed a previously applied protocol^21^. An initial energy minimization was done to reduce steric tensions in the starting structures. The minimization was subdivided into three steps. First, only water molecules were minimized whereas all other atoms were restrained with a force of 10 kcal ⋅ mol^−1^ ⋅ Å^−2^. Second, the Cl^−^ ion and the protein hydrogen atoms were minimized as well while the rest was fixed with the same force again. Finally, an unrestrained minimization of the complete system was carried out. Each of the minimization steps comprised 2, 500 steps of *steepest descent* as well as 2, 500 steps of the *conjugate gradient* algorithm. The following equilibration was also divided into three parts with a time step of 2 fs: During the first 0.1 ns, the system was heated from 10 K to 310 K while the protein and ligand atoms as well as the Ca^2+^ ions were restrained with a force of 5 kcal ⋅ mol^−1^ ⋅ Å^−2^. At constant pressure and temperature, only the Cα atoms were kept fixed for another 0.4 ns. Then, all atoms were equilibrated for 0.5 ns in the last step. Minimization and equilibration were carried out on CPUs. In contrast, the production runs were performed using pmemd.CUDA on GPUs^22^. Each production phase comprised 300 ns with a time step of 2 fs, a constant temperature of 310 K regulated by a Berendsen thermostat^23^ and a constant pressure of 1 bar. The SHAKE algorithm^24^ was applied on bonds with hydrogen atoms both in the equilibration and production phase.

Trajectory analysis (radius of gyration, atomic distances, van der Waals energies, analysis of root-mean-square fluctuations) was carried out using the Amber tool cpptraj^25^. Contacts were determined with an in-house Perl script parsing the trajectory and assigning contacts based on a distance criterion of ≤5 Å between any pair of atoms. Plots were created with Gnuplot^26^ and T_E_Xlive^27^, structure images with UCSF Chimera^17^.

### Data availability

All relevant data generated or analyzed during this study are included in this published article (and its Supplementary Information files).

## Results and Discussion

### Overall properties of the Mincle-glycolipid interaction

**Figure 2:**
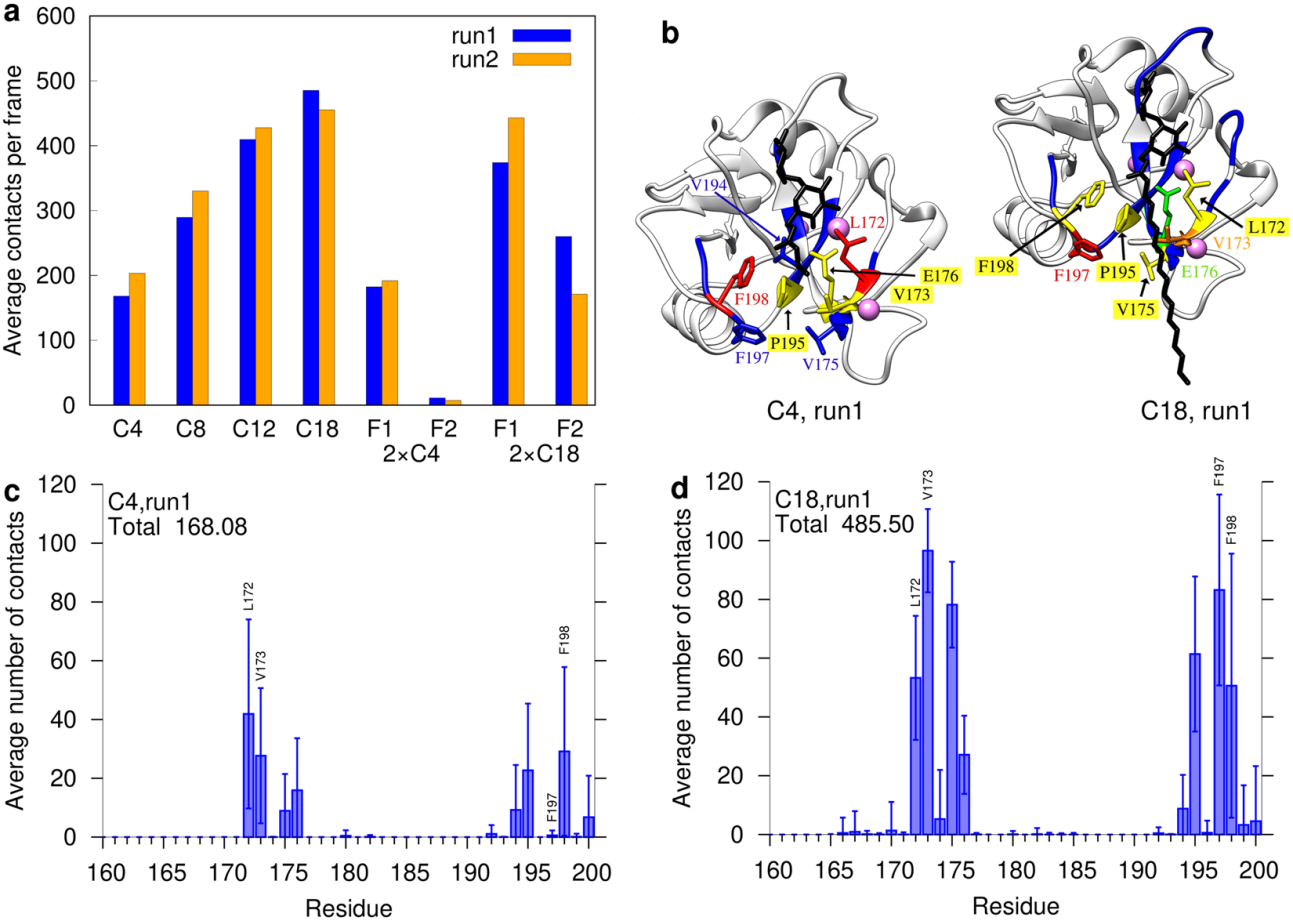
Contacts between Mincle and the acyl chains. (**a**) Average number of contacts per system and acyl chain; (**b**) Initial structures of the C4 and C18 monoesters in complex with Mincle. Residues are colored according to their average number of contacts with the acyl chain: red > orange > yellow > green > blue. (**c**,**d**) Contacts per Mincle residue for (**c**) C4 and (**d**) C18 monoesters in run 1. For each Mincle residue, the average number of contacts (±standard deviation) to the acyl chains is plotted. Some bars representing key residues are directly labeled in the plot. The data for the remaining mono- and diesters of run1 and for run2 is shown in the Supplementary Figures S6–S9.

First, we investigated the total number of contacts formed between protein and ligand. We noted that the trehalose moiety remains very tightly bound in all simulations and that the number of trehalose-protein contacts is virtually identical in all systems investigated. Therefore, all subsequent analyses focused on the acyl sidechains, which differed between the various ligands. We observed for the monoesters that the overall number of contacts increased almost linearly with the length of the acyl chain from C4 to C12 (Fig. 2a). Despite the limited extension of the hydrophobic groove, even acyl chains with 18 carbon atoms (C18) showed still an increased number of contacts compared to C12 fatty acids. However, the increase was less pronounced compared to the shorter acyl chains (C4 to C12) (Table 2). For each of the systems investigated, we also noted that the results from the two independent simulation runs were quite similar. This indicates that the number of contacts is not critically affected by the initial ligand conformation used as starting point for the simulations.

**Table 2:** Ratio of contact numbers between Mincle and the acyl chains for the systems on the *y* axis and the systems on the *x* axis as an average over both runs.

Systems (*y*/*x*)	C4	C8	C12	C18	2 × C4	2 × C18
C4	1					
C8	1.59	1				
C12	2.25	1.42	1			
C18	2.53	1.60	1.12	1		
2 × C4	1.06	0.67	0.47	0.42	1	
2 × C18	3.36	2.12	1.49	1.33	3.18	1

In case of the 2 × C4 diester, the first fatty acid (F1) showed a similar number of contacts as the C4 monoester, whereas the contacts formed by the second fatty acid (F2) were almost negligible (<20 on average) (Fig. 2a). In contrast to the short 2 × C4 diester, the second fatty acid of the longer 2 × C18 diester formed approximately 200 additional contacts.

### Characterization of the Mincle binding site

The next step was to examine which protein residues were contacted predominantly by the fatty acids. Based on mutagenesis experiments Feinberg *et al*. proposed Leu172, Val173, Phe197, and Phe198, which line the hydrophobic groove^7^, as key interaction partners of bound acyl chains (Supplementary Figure S19a). Our simulations confirm that these four residues were actually responsible for most of the contacts (Fig. 2b–d). Adjacent residues like Val175, Glu176, Val194 or Pro195 also formed a significant number of contacts, and there are ≈20 additional residues, for which only a very small number of contacts was detected (shown in blue in Supplementary Figure S19a). All these interacting residues are rather well conserved between bovine Mincle investigated in the present study and human Mincle (Supplementary Figure S19b). It is interesting to note that the set of interacting residues is highly similar for different acyl chain length (Fig. 2c,d; Supplementary Figures S6–S9). This indicates that longer acyl chains rather form more contacts to the hydrophobic groove, than interacting with additional other surface patches. This is even the case for 2 × C18, containing two long fatty acid sidechains (Supplementary Figures S8c,d and S9c,d).

**Figure 3:**
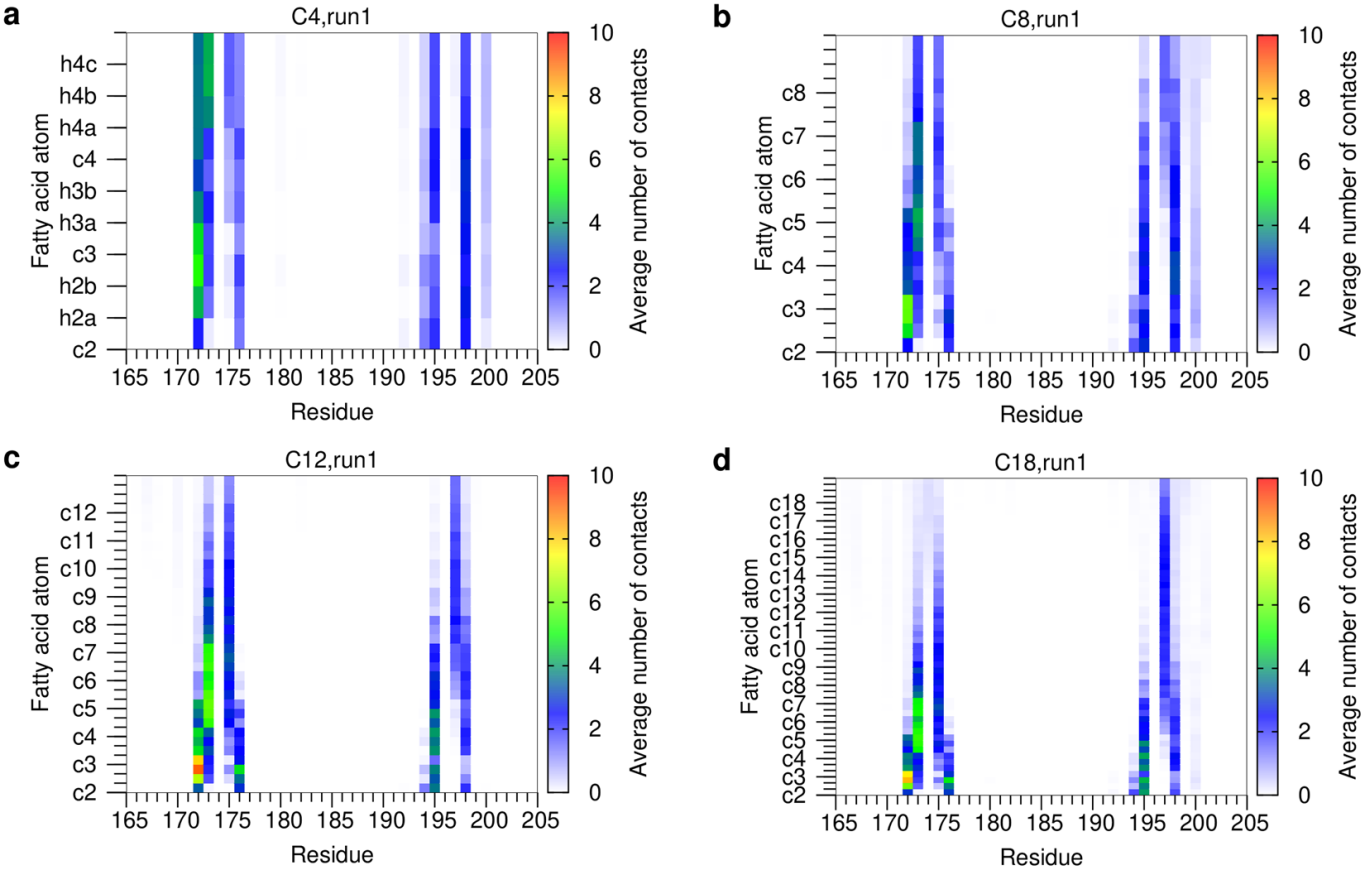
Contacts per Mincle residue and acyl chain atom for monoesters (run 1). (**a**) C4; (**b**) C8; (**c**) C12; (**d**) C18. For each Mincle residue (*x*-axis), the contacts with the individual atoms of the acyl sidechains (*y*-axis) are plotted. The y-axis starts with the c2 atom and leads upwards to more distal CH_2_ groups. For every CH_2_ group, first the carbon atom and then the two attached hydrogen atoms are shown resulting in three fields in total; in case of the terminal CH_3_ group, the carbon atom is followed by three hydrogen atoms. All these atoms are explicitly labeled in panel (a). For clarity, only the carbon atoms of the acyl chain are labelled on the *y*-axis of the remaining panels, and the bars displaying the contacts of the connected hydrogen atoms are indicated as minor ticks. The contact map is colored according to the average number of contacts formed. The data for run2 is shown in Supplementary Figure S12.

To dissect, which of the fatty acid carbons is responsible for contact formation, we calculated the number of protein contacts separately for each atom of the chain. In all simulations of the monoesters, L172 represents a key interaction site that is contacted by the carbon atoms located proximal to the trehalose moiety (i.e. c2–c4) (Fig. 3). Other interacting residues like V173, V175, or F198 can be contacted by a wide range of different carbon atoms along the acyl chain (≈c4–c12) indicating that there is no rigid binding geometry (Fig. 3). Interestingly, the key interacting residue F197 preferentially interacts with the distal carbon atoms of the fatty acid (≈c6–c18; Fig. 3). Thus, this interaction offers an explanation for the experimentally observed increase in binding affinity for longer fatty acids^8,10,28^.

**Figure 4:**
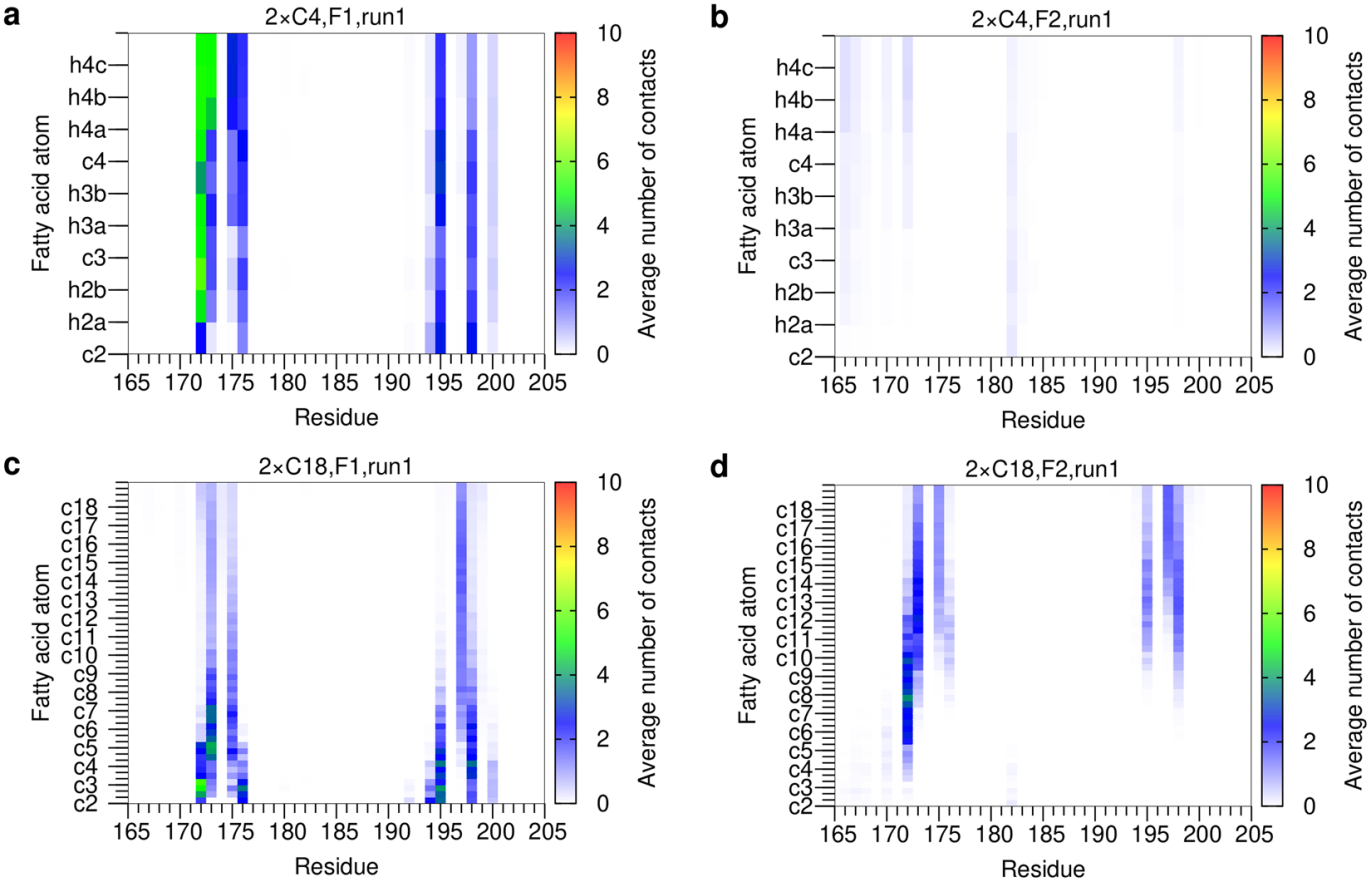
Contacts per Mincle residue and acyl chain atom for diesters (run 1). (**a**) 2 × C4, F1 acyl chain; (**b**) 2 × C4, F2 acyl chain; (**c**) 2 × C18, F1 acyl chain; (**d**) 2 × C18, F2 acyl chain. For each Mincle residue (*x*-axis), the contacts with the individual atoms of the acyl sidechains (*y*-axis) are plotted. The *y*-axis starts with the c2 atom and leads upwards to more distal CH_2_ groups. For every CH_2_ group, first the carbon atom and then the two attached hydrogen atoms are shown resulting in three fields in total; in case of the terminal CH_3_ group, the carbon atom is followed by three hydrogen atoms. All these atoms are explicitly labeled in panel (a). For clarity, only the carbon atoms of the acyl chain are labelled on the y-axis of the remaining panels, and the bars displaying the contacts of the connected hydrogen atoms are indicated as minor ticks. The contact map is colored according to the average number of contacts formed. The data for run2 is shown in Supplementary Figure S13.

For the diesters (Fig. 4), the contacts formed by the first fatty acid residue (F1) are highly similar to those observed in the respective monoesters. For the second fatty acid residue (F2) of 2 × C18 only the distal carbons are capable of forming interactions with Mincle (Fig. 4d). Consequently, the short F2 sidechain of the 2 × C4 ligand forms almost no interactions with Mincle (Fig. 4b).

### Dynamics of the Mincle-glycolipid interactions

**Figure 5:**
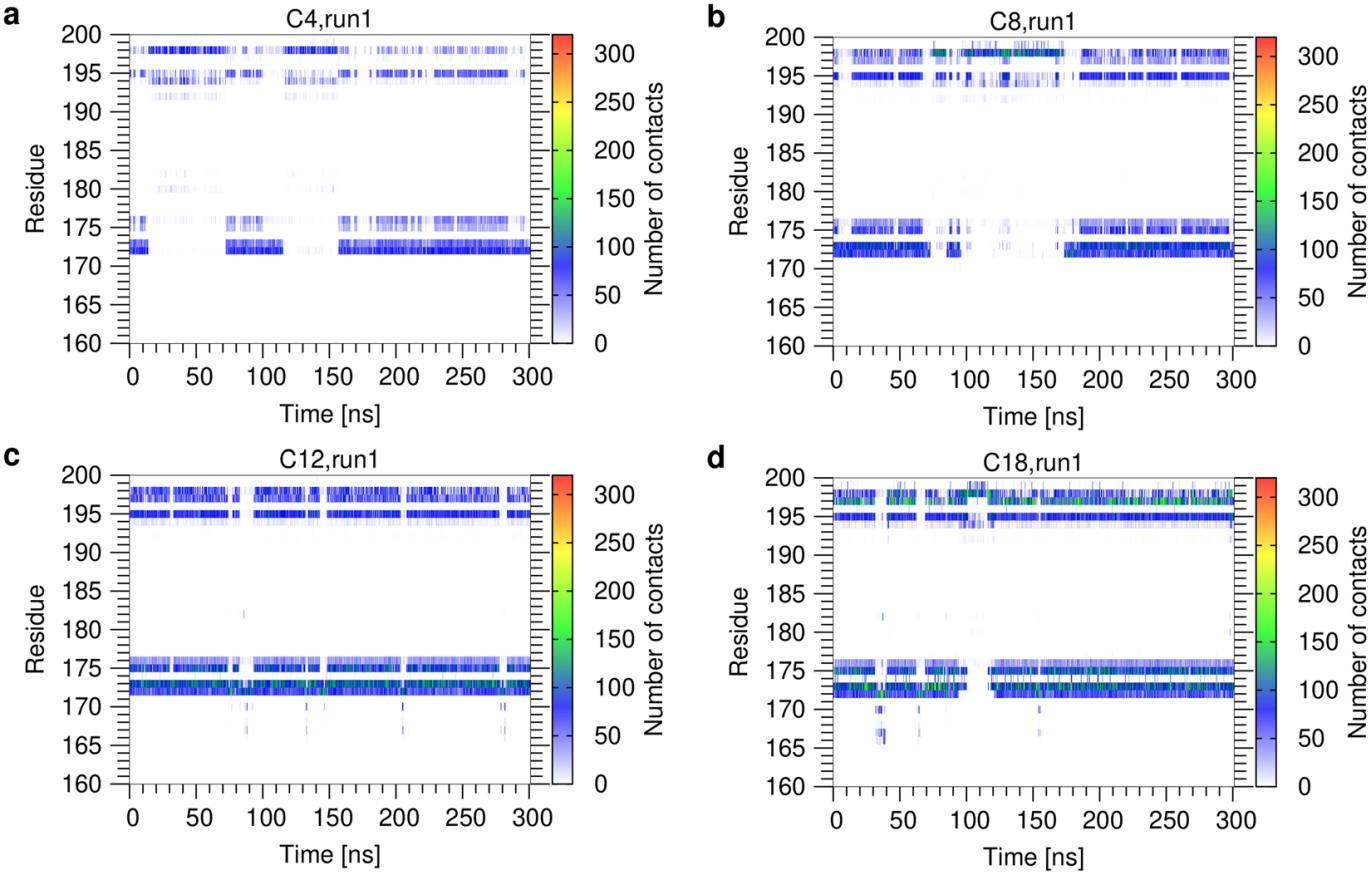
Contacts per Mincle residue as a function of simulation time for monoesters (run 1). (**a**) C4; (**b**) C8; (**c**) C12; (**d**) C18. Contacts were monitored for the individual residues of Mincle (shown on the y-axis) over the simulation time. The contact map is colored according to the number of contacts formed. The data for run2 is shown in Supplementary Figure S14.

**Figure 6:**
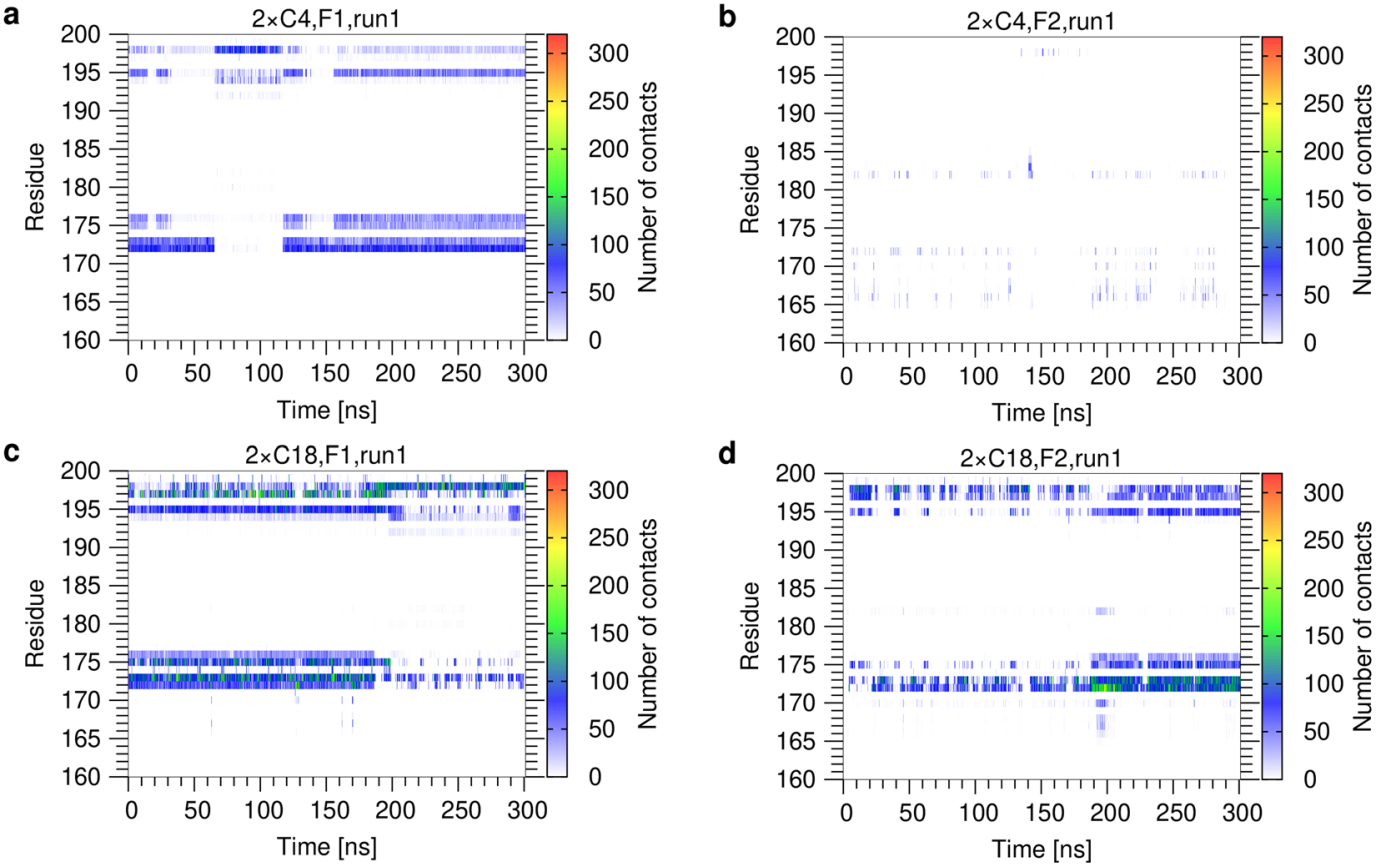
Contacts per Mincle residue as a function of simulation time for diesters (run 1). (**a**) 2 × C4, F1 acyl chain; (**b**) 2 × C4, F2 acyl chain; (**c**) 2 × C18, F1 acyl chain; (**d**) 2 × C18, F2 acyl chain. Contacts were monitored for the individual residues of Mincle (shown on the y-axis) over the simulation time. The contact map is colored according to the number of contacts formed. The data for run2 is shown in Supplementary Figure S15.

The acyl chains do not interact with Mincle in a single defined orientation, but remain generally very flexible and sample a wide distribution of different conformations from extended to compact structures (Supplementary Figures S10, S11). The dynamic nature of the Mincle-glycolipid interaction can be seen in more detail in a plot of contacts versus simulation time for the individual residues (Figs 5 and 6). For the monoesters (Fig. 5), these plots confirm the previous observation that short and long acyl sidechains contact the same set of residues on the Mincle surface. In addition, the plots reveal that these interactions are formed for a longer portion of the simulation time with increasing chain length (Fig. 5). The average contact lifetime of the Mincle-glycolipid interaction is less than 10 ns for the short acyl esters and increases up to 20 ns for the C18-chains. However, despite being transient, interactions with the residues of the hydrophobic groove were observed for most periods of the simulation time (Fig. 5). So, the contacts formed and broke repeatedly with the same residues. From this observation we also concluded that conventional MD simulations are sufficient for sampling of the conformational space and refrained from the application of enhanced sampling methods (e.g. replica-exchange MD).

**Figure 7:**
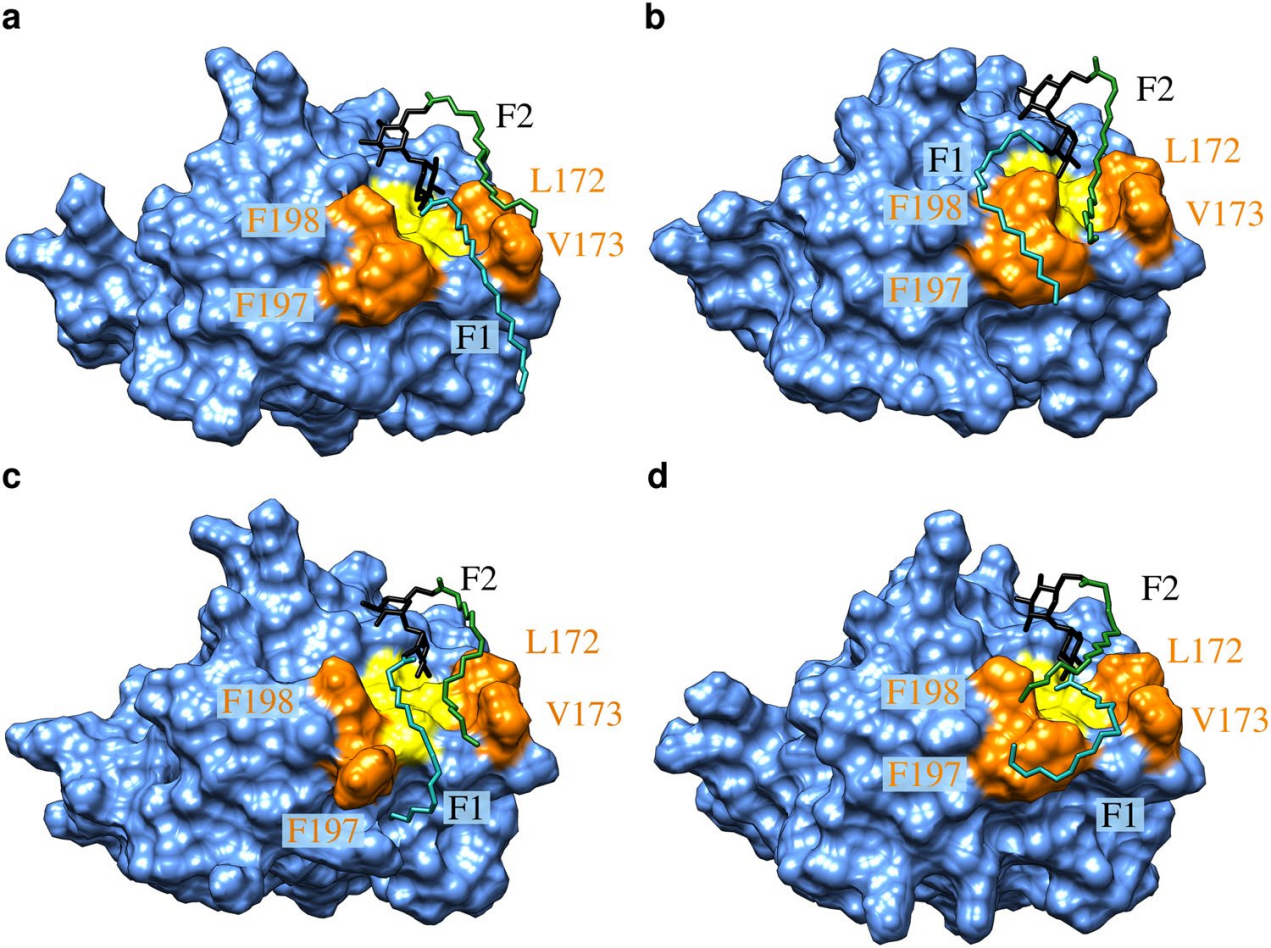
Representative binding modes of the 2 × C18 diester to Mincle. Mincle is shown in blue surface presentation with the hydrophobic groove in yellow. Hydrophobic residues lining the groove are shown in orange and are labelled. The glycolipid is shown in stick presentation with the trehalose, F1 and F2 chains are colored in black, cyan, and green, respectively. See text for a detailed description of the binding modes (**a**–**d**) and the Supplementary movie for the dynamic nature of the interaction.

For the 2 × C4 diester (Fig. 6a,b), the dynamics of the contacts formed by the F1 chain is highly similar to that of the monoester (Fig. 5a), whereas the F2 chain forms only very weak and transient interactions. In contrast, the long F2 chain of the 2 × C18 diester actively participates in binding. The time dependent analysis of the 2 × C18 diester (Fig. 6c,d) revealed a rather complex interplay between both acyl chains, which was investigated in more detail on a structural level (Fig. 7; Supplementary movie). During the first half of the simulation time, F1 is frequently located within the hydrophobic groove, whereas F2 is located at the edge of the groove and interacts with L172/V173 or F197/F198 from the outside (Fig. 7a). An alternative arrangement is found during the last 100 ns of the simulation, in which F2 represents the major interacting residue in the hydrophobic groove and F1 interacts mainly with F197/F198 from the outside (Fig. 7b). In addition to these situations, in which only one of the acyl chains is inserted into the groove, we also detected conformations in which both acyl chains are inserted at the same time (Fig. 7c). Interestingly, this binding mode is rather rarely observed and can be accompanied by transient changes of the sidechain conformation of the adjacent residues. These observations suggest that the hydrophobic groove in Mincle is not sufficiently wide to allow a stable insertion of two acyl chains at the same time.

In addition, even conformations having only one chain inserted into the hydrophobic groove can exhibit additional modes of stabilization as exemplarily shown in Fig. 7d. In this conformation, F1 is inserted inside the groove and the distal carbons, which extend the dimensions of the groove, adopt a kinked conformation and interact with the phenylalanines from the outside. Such kinks might be even more favored by the presence of a cyclopropyl ring in mycolic acid sidechains as found in TDM. In our simulation, the F2 chain forms a lid on the groove and interacts both with the key hydrophobic residues and also with the F1 sidechain. Like for the F1 chain, the distal carbons are involved in this interaction thus offering an explanation for the tighter binding of long acyl esters. The latter conformation was also found to represent a frequent conformation in the second simulation run of 2 × C18 (Supplementary Figure S15). The observation that F2 does not completely replace F1 in the grove during the simulation time further supports the conformational stability of this arrangement.

The data above for 2 × C18 demonstrates that a second acyl chain can form additional contacts with Mincle if it is sufficiently long. However, since both chains in part compete with the favorable interaction in the hydrophobic groove, the number of contacts detected for the F1 chain becomes slightly decreased compared to the C18 monoester (Fig. 2a). Depending on the degree of competition for groove binding, the magnitude of this effect differs slightly between both simulation runs (Fig. 2a). However, this loss of contacts of the F1 chain is more than compensated by the additional interactions formed by the F2 chain, leading to a stronger interaction of the 2 × C18 diester compared to the C18 monoester.

**Figure 8:**
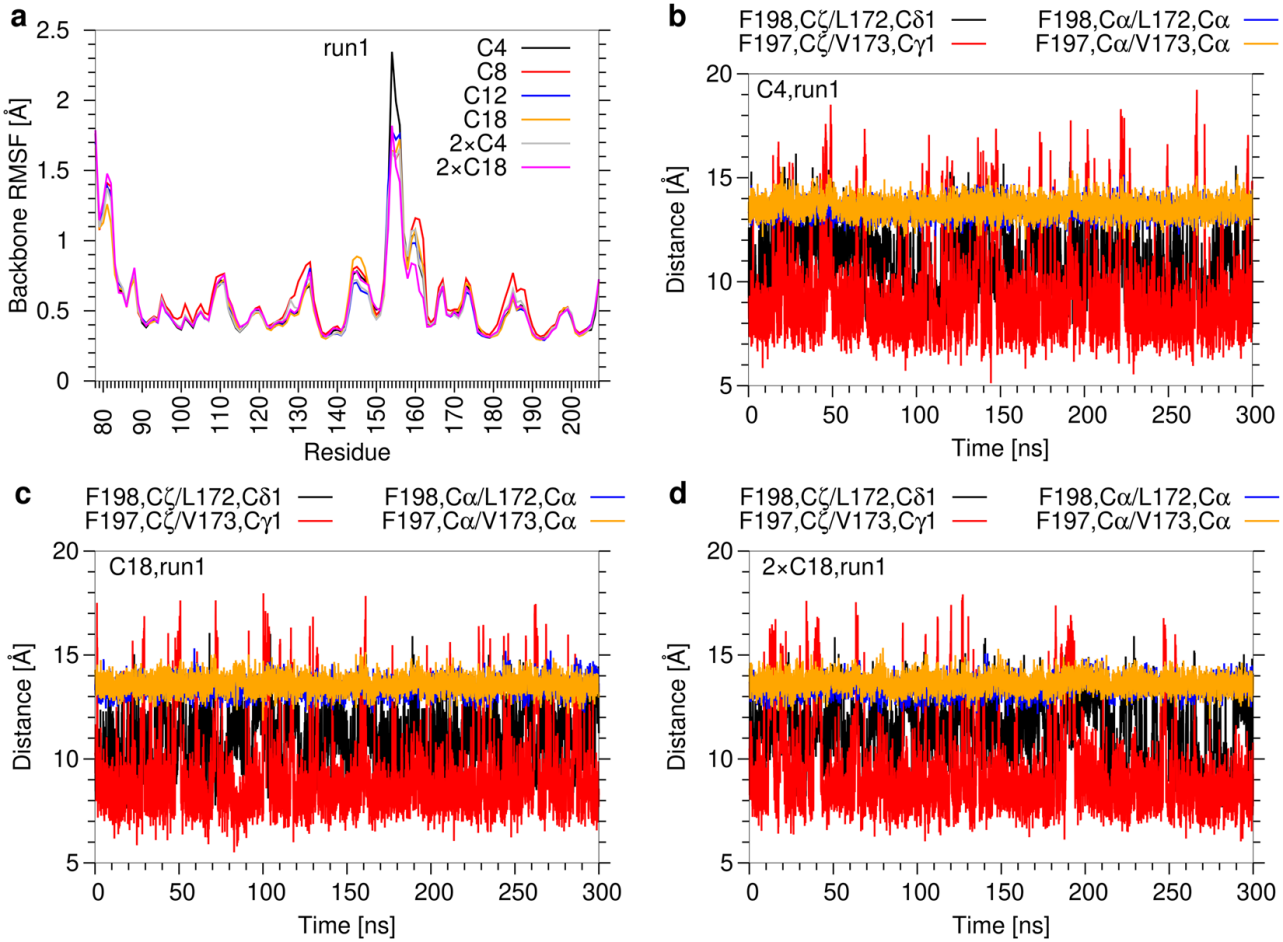
Conformational stability of Mincle. (**a**) Backbone root-mean-square fluctuations per Mincle residue (run1) for the complexes with different glycolipids. (**b**–**d**) Width of the hydrophobic groove (run1) for (**b**) C4; (**c**) C18; (**d**) 2 × C18 as ligand. Distances between the most distal side chain atoms or between the Cα-atoms were measured for residue pairs lining the hydrophobic groove on opposite sides (i.e. F198-L172 and F197-V173 pairs). The data for the remaining ligands of run1 and for run2 is shown in Supplementary Figures S16–S18.

The analyses above reveal a significant variability in the recognition of the hydrophobic groove and that two acyl sidechains are hardly accommodated in the groove at the same time. This prompted us to investigate the dynamics of Mincle itself and the geometric properties of the hydrophobic groove in more detail. The root-mean-square fluctuations of the Mincle backbone atoms (Fig. 8a) indicate the highest flexibility in the region of the truncated N-terminus and in a loop between residues 145–155. In contrast, the regions which form contacts with the acyl chains (i.e. residues 170–175 and 195–200) showed much smaller fluctuations of 0.5–0.75 Å. Moreover, no systematic differences could be detected for acyl chains of different lengths or between mono- and diesters. An inspection of the backbone atomic distances between the residues on opposite sides of the hydrophobic groove (F198-L172, F197-V173) confirms that there are only minor fluctuations and no conformational rearrangements (Fig. 8). The distances between the sidechains of the respective residue pairs showed larger fluctuations between 8 and 16 Å suggesting that there is a certain degree of conformational freedom.

However, we did not observe an increase in these sidechain distances for 2 × C18 diester compared to the C18 monoester indicating that the presence of the second acyl chain has only a marginal effect on the structure of the hydrophobic groove (Fig. 8c,d). Taken together, these observations suggest that the glycolipid binding site of Mincle exhibits a rather high conformational stability, which hampers the accommodation of two acyl chains into the hydrophobic groove at the same time.

### Energetics of the Mincle-glycolipid interaction

**Figure 9:**
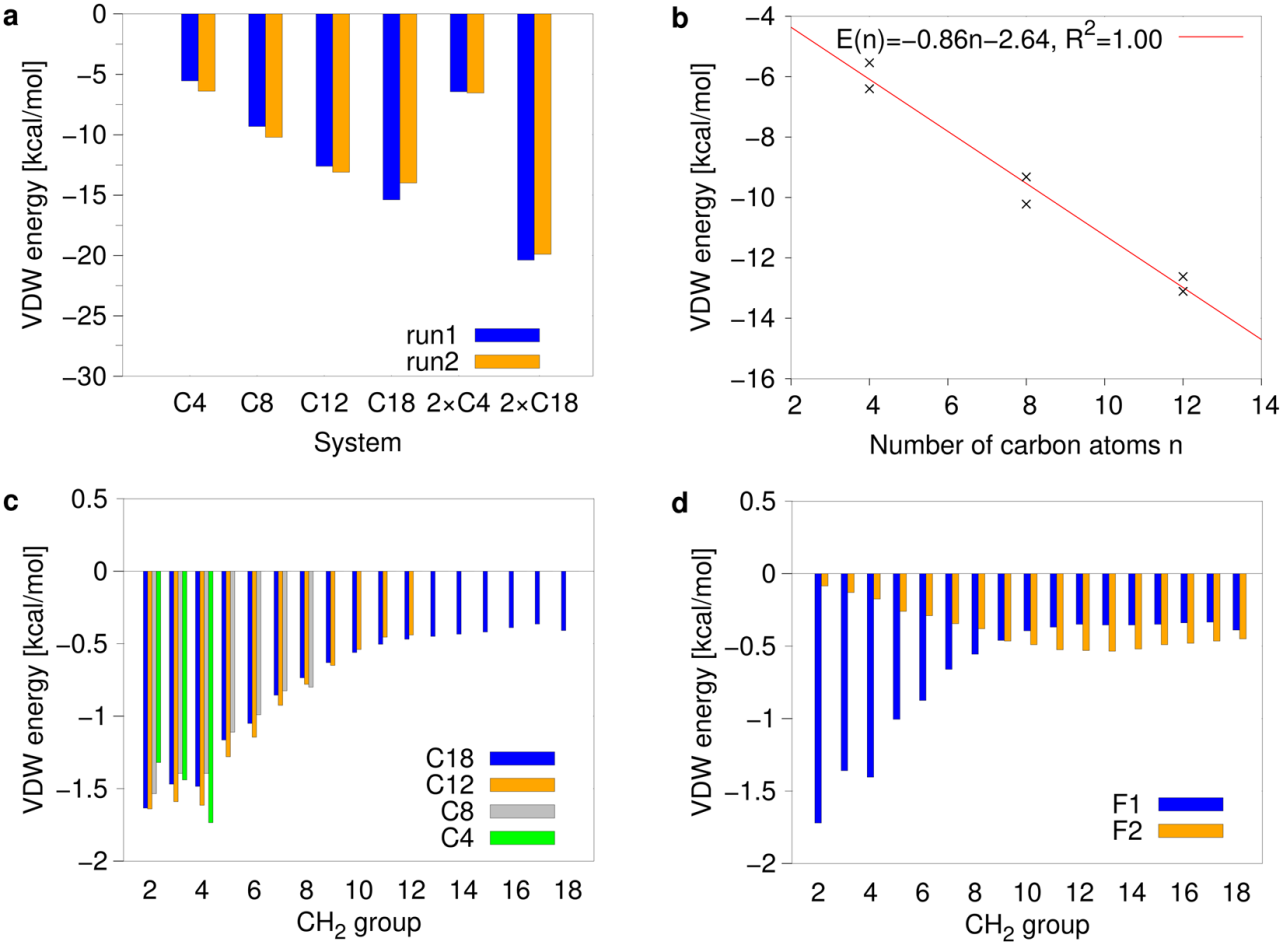
Van derWaals interaction energy between Mincle and ligands. (**a**) Average interaction energy observed for the acyl moieties of different ligands. (**b**) Correlation between the number of carbon atoms and the interaction energy. The fit was performed for the C4 to C12 monoesters from both simulation runs. (**c**,**d**) Interaction energy of the individual CH_2_ groups of the acyl chains in (**c**) monoesters and (**d**) the 2 × C18 diester. Energies were averaged over both simulation runs.

After the analysis of structural properties, we compared the binding affinity of the ligands. Since the glycolipids investigated differ only in the length and number of the acyl sidechains attached, our analysis focused on the lipid moiety. Due to the hydrophobic nature of the lipid sidechains, we considered the van der Waals interaction energy as a meaningful measure for the strength of the interaction. As already expected from the number of contacts, the interaction energies of the monoesters become more favourable with increasing length of the acyl chain (Table 3, Fig. 9a). A more detailed analysis (Fig. 9b) reveals that there is a linear increase in interaction energy for the acyl chain lengths C4, C8, C12. This finding is remarkable, because the length of the hydrophobic groove only allows to accommodate approximately six CH_2_ groups in an extended conformation^6^. However, due to the dynamic nature of the interaction (Figs 5, 6) and existence of non-extended acyl conformations (Fig. 7, Supplementary Figs S10, S11, Supplementary movie), more distal methylene groups can still efficiently contribute to the interaction with Mincle. For the longer C18 monoesters, the increase in binding energy is less pronounced (Table 3, Fig. 9a). An energy decomposition for the individual CH_2_ groups (Fig. 9c) reveals that the groups 2–4 form the strongest interactions. For the groups 5–12 there is a gradual decrease in interaction energy, which levels off at a value of −0.4 to −0.5 kcal/mol for the more distal CH_2_ groups. This indicates that distal CH_2_ groups can still contribute to binding; however, one has to keep in mind that the free energy of binding is also affected by entropic contributions, which comprise both a loss of conformational flexibility in the acyl chain and a release of bound water molecules (“hydrophobic effect”). Consequently, the sum of these opposite entropic contributions may affect the free energy of binding in a favorable or an unfavorable fashion.

**Table 3:** Van der Waals energies between Mincle and the fatty acids. Averages and standard deviations for the whole simulation time.

System	Van der Waals energy [kcal/mol]	
	run1	run2
C4	−5.54 ± 2.30	−6.40 ± 2.11
C8	−9.32 ± 3.31	−10.22 ± 2.77
C12	−12.62 ± 3.08	−13.11 ± 2.48
C18	−15.40 ± 3.64	−14.28 ± 4.48
2 × C4, F1	−5.87 ± 1.94	−6.13 ± 2.18
2 × C4, F2	−0.57 ± 0.87	−0.42 ± 0.70
2 × C18, F1	−12.42 ± 4.26	−14.26 ± 3.20
2 × C18, F2	−7.97 ± 4.08	−5.64 ± 2.54

For the 2 × C4 diester, the energetic contribution of the second acyl chain is very small and the total van der Waals interaction energy is increased by less than 1 kcal/mol larger compared to the C4 monoester (Table 3, Fig. 9a). For the 2 × C18 diester, particularly the more distal CH_2_ groups of the second acyl chain contribute to binding (Fig. 9d), which is in line with the geometric properties of the complex, in which only distal atoms of the F2 chain are able to contact the hydrophobic groove (Fig. 7). For groups 10–18, the energetic contribution of F2 to binding is even larger than that of the F1 chain. In total, the van der Waals binding energy of the 2 × C18 diester is 36% higher than that of the C18 monoester (Fig. 9a).

### Link to experimental data

The present MD study sheds light onto the structure, dynamics and energetics of the Mincle-glycolipid interaction. This information offers a molecular explanation for several experimental findings as described below:

Our simulations revealed a remarkably high flexibility of the acyl side chains even upon interaction with Mincle (Figs 5–7). This finding offers an explanation for the fact that in a complex crystal structure of Mincle with trehalose monobutyrate only the proximal parts of the acyl chain are resolved^7^ and that there exist no experimental structures for complexes with longer acyl chains to date. Our studies suggest the hydrophobic groove represents the sole interaction site confirming the key role of the hydrophobic residues L172, V173, F197, and F198 for ligand binding, which was previously deduced from site-directed mutagenesis^6^.

A second finding from the MD study was a linear increase of the van der Waals interaction energy for the shorter C4-C12 monoesters (Fig. 9b). The same correlation was observed in several experimental studies^6,7,9^ suggesting that the present MD simulations are capable of adequately reflecting the binding mode for this group of ligands. For the 2 × C4 diester, the simulations suggest only a minor increase of binding affinity compared to the C4 monoester. The experimentally observed 3.5-fold increase in affinity^9^ is slightly larger supporting the previous notion^6,7^ that the avidity of the diesters favours binding (see details below).

For the longer acyl esters, there exists no quantitative experimental binding data, and their potency has instead been measured by monitoring NO synthesis, GCSF or Interleukin production^8,28^. These studies indicate that long acyl chains (C14-C22; depending on the experimental setup) are required for immune activation^8,10,28^. In addition, most experimental assays detected a significant biological effect only for diesters^8,28^. The present simulations indicate that both the length of the acyl chain and the presence of a second long chain enhance binding affinity. However, there is evidence that the strength of the interaction measured in the present setup is not the only factor that is responsible for the biological activity of ligands. One factor, that has been previously discussed to enhance binding of diesters is avidity^6,7^. The avidity results from the fact that symmetric trehalose disaccharides can bind in two opposite orientations to Mincle. In case of the diesters, each of these orientations will allow to place one acyl chain in the immediate vicinity of the hydrophobic groove (corresponding to the R1 position in Fig. 1). Avidity becomes only effective after dissociation and re-binding of the ligand, which occur on time scales not accessible to our MD simulations. A second factor that might affect biological activity is the solubility of the glycolipids and also their tendency to form aggregates or micelle-like structures. Finally, it was speculated that the enhanced immune activity observed for diesters could also be due to binding of the second fatty acid to another receptor molecule^28,29^. Mincle forms heterodimers with the homologous macrophage C-type lectin (MCL)^30,31^ that has a hydrophobic groove similar to Mincle. Thus diesters might either act as an intra-dimeric link stabilizing the heterodimer or even link separate heterodimers^29^. Although the features responsible for a biological activity of ligands are not fully understood to date, the experimental data strongly suggests that long acyl side chains are a prerequisite for biological activity^8,10,28^.

The present study sheds light onto the molecular details of the interaction between long acyl chains and Mincle. Our simulations show that binding of both acyl chains occurs almost exclusively with the residues forming the hydrophobic cleft. Despite the limited extension of this cleft, the dynamic nature of the interaction and the conformational flexibility of the acyl chains allow that also distal CH_2_ groups do contribute favorably to the interaction. These effects are not only observed for the F1 chain, but also for the F2 chain, which is attached on the opposite site of the hydrophobic cleft and has to fold back over the trehalose moiety in order to interact with the hydrophobic groove (Fig. 7). This structural information is considered helpful for the design of more affine glycolipid ligands in the future, that may facilitate the development of novel vaccines for a robust induction of cellular immune responses in humans.

## Electronic supplementary material

Supplementary Information

Dataset 1

Dataset 2

Movie of the mincle-glycolipid complex
