# Supplementary material for: Interaction of Glycolipids with the Macrophage Surface Receptor Mincle – a Systematic Molecular Dynamics Study
Source: Sci Rep. 2018 Mar 29;8:5374. doi: 10.1038/s41598-018-23624-8 (PMC5876379; doi:10.1038/s41598-018-23624-8)
Supplement: Supplementary file 1 — Supplementary Information [file 41598_2018_23624_MOESM1_ESM.pdf]

Supplementary Information

Force Field Parameter Generation and Complementary Figures

Christian A. Söldner, Anselm H. C. Horn, Heinrich Sticht

Contents

1 Supplementary Methods – Atom Types and Charges for Trehalose Acyl Esters 1
1.1 Carbohydrate Residues 1
1.2 Acyl Chains 3
1.3 AMBER prep files 5

2 Supplementary Results – Complementary Figures 6
Supplementary Fig. S6 6
Supplementary Fig. S7 7
Supplementary Fig. S8 8
Supplementary Fig. S9 9
Supplementary Fig. S10 10
Supplementary Fig. S11 10
Supplementary Fig. S12 11
Supplementary Fig. S13 12
Supplementary Fig. S14 13
Supplementary Fig. S15 14
Supplementary Fig. S16 14
Supplementary Fig. S17 15
Supplementary Fig. S18 16
Supplementary Fig. S19 17

3 References 18

1 Supplementary Methods – Atom Types and Charges for Trehalose Acyl Esters

The trehalose acyl monoesters and diesters in our MD simulations were assigned atom types and force field parameters of the GLYCAM06j-1 force field [1]. This force field, which is specifically designed for a description of carbohydrates, has already been applied in previous studies for the simulation of fatty acids [2] and for systems, where glycosids and acyl chains are directly linked together [3, 4].

1.1 Carbohydrate Residues

Trehalose (1-α-Glucopyranosyl-1-α-Glucopyranosid) is a disaccharide consisting of two glucose moieties, which are linked via an α, α'-1, 1-glycosidic bond (structure formula in Supplementary Fig. S1).

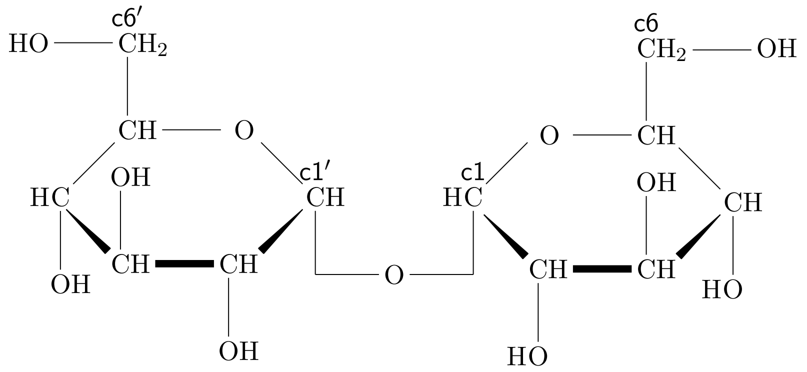

Supplementary Fig. S1. *Structure formula of trehalose.*

The GLYCAM06 force field provides predefined residues for some α-D-Glucopyranose isomers with free valences at different positions allowing thereby connectivity to other residues in a modular concept. Examples for already available residues are as shown in Supplementary Fig. S2:

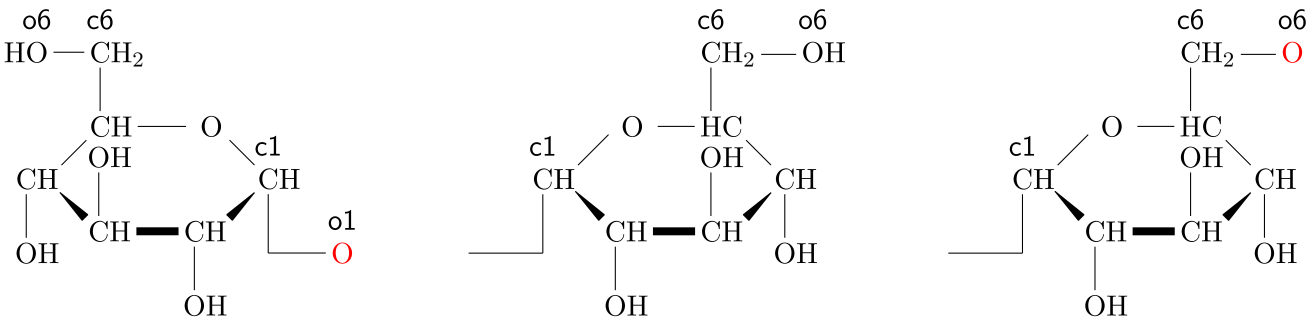

(a) *1GA* $(q = -0.194\,e)$ ***(b)*** *0GA* $(q = +0.194\,e)$ ***(c)*** *6GA* $(q = 0)$

Supplementary Fig. S2. *Predefined Glucose residues in the* GLYCAM06 *force field and their respective total charges. Free valences are coloured in red.*

In order to construct trehalose, they can be combined as

(a) 1GA + 0GA, if no acyl chains are present. The partial charges of both residues add up to neutral.

(b) 1GA + 6GA, if a connection to an acyl chain at the o6 atom is desired. This path was chosen for the trehalose-6-monoesters C4, C8, C12 and C18. The partial charges of both residues add up to $-0.194\,e$ so that the acyl chain needs a total charge of $+0.194\,e$ for electrical neutrality.

However, the residues predefined in GLYCAM are not sufficient for the construction of trehalose-6,6′-diesters, because no glucose residue exists that has an oxygen, but no hydrogen atom and therefore free valences at both c1 and c6 position. Therefore, we constructed a new residue CGA (Supplementary Fig. S3), based on the template 1GA using the AMBER14 [5] version of the `xleap` program.

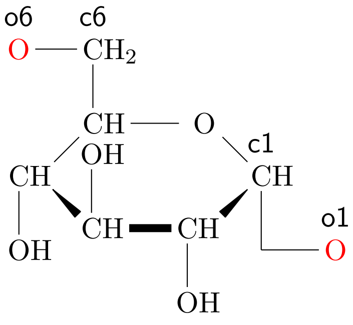

Supplementary Fig. S3. *CGA* $(q = -0.388\,e)$

The GLYCAM06j-1 residues were loaded, and 1GA was opened for editing:

```
source leaprc.GLYCAM_06j-1
edit 1GA
```

In the graphical user interface, the hydrogen atom connected to o6 (leap atom name H6O) was deleted. Then, the modified residue was written to an AMBER prep file with the command

```
saveamberprep 1GA CGA.prep
```

In the new prep file, we changed the residue label from 1GA to CGA. In analogy to the conventions of the GLYCAM06 force field, the atom type of the former hydroxy oxygen atom o6 was changed from `Oh` to `Os`. The predefined GLYCAM residues comprise in total eight different isomers of α-D-Glucopyranose, which have a free valence at the o6 oxygen atom, and another eight, which have a complete hydroxy group at this position. Although these residues differ in the overall number of free valences, a detailed comparison shows that all residues without the hydrogen atom have the same partial charge of $-0.458\,e$ at the o6 atom, whereas those with hydrogen atom have a charge of $-0.682\,e$. The charge of the adjacent carbon atoms is not influenced by the existence of the hydrogen. Hence, we changed the charge of the o6 atom to $-0.458\,e$. In consequence, the total charge of the residue had to be modified to $-0.388\,e$.

The new residue CGA was used in the simulations of our trehalose diacylesters 2×C4 and 2×C18 in the combination CGA + 6GA leading to an overall carbohydrate charge of $-0.388\,e$. By adding two fatty acids with a charge of each $+0.194\,e$, electrically neutral diesters could be created.

For the setup of our simulations, we used the trehalose atom coordinates, that were present in the PDB file `4ZRV`. Therefore, the residue identifiers of these atoms hat to be corrected to the respective GLYCAM residue 1GA, 6GA or CGA. Moreover, the atom names were changed to fit the atom names used in the residue templates. Then, we created our topology and the initial AMBER coordinate file using `tleap`. The prep file was loaded using the command `loadamberprep`. One last necessary step was to define the glycosidic bond between the two residues with the two sugar residues and the ester bonds between sugar and acyl chains by means of the `bond` command.

1.2 Acyl Chains

1.2.1 Generation of 3D structures

We created 3D structures and AMBER prep files for acyl chains with $n \in \{2, 4, 6, 8, 10, 12, 14, 16, 18, 20, 22\}$ carbon atoms, which we call `F02`, `F04`, ..., `F22`. Although not all these chain length were necessary for the present MD simulations, we generated a complete set covering a large variety of chain lengths in order to facilitate future studies. In order to make them easily combinable with the glucose residues of the trehalose, our acyl chain residues begin at the carbonyl carbon atom c1 and do not contain the acidic hydroxy group. The ester oxygen atom is in our setup still part of the sugar moiety (Supplementary Fig. S4). However, to allow for a RESP/ESP fit of charges, we created also PDB files of complete alkyl acids including the acidic hydroxy group. All initial PDB files were generated with AVOGADRO 1.1 [6] by drawing the necessary atoms and adjusting inter-atomic distances, angles and torsions.

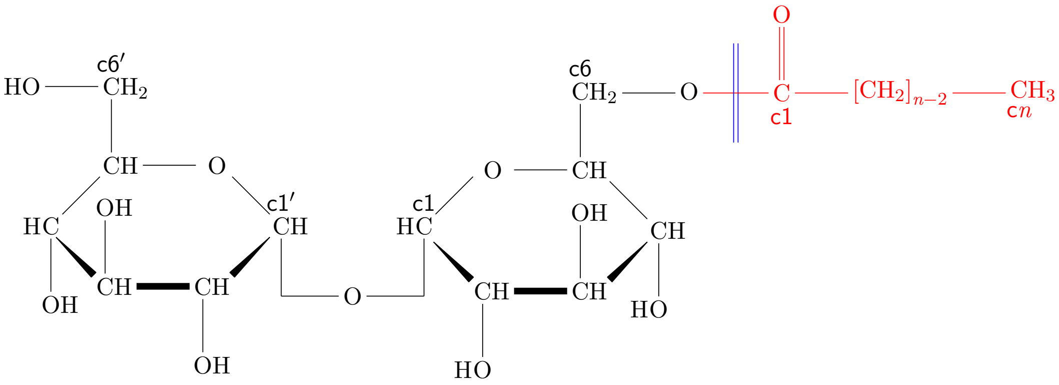

Supplementary Fig. S4. *Subdivision of sugar and acyl chain residues in our setup.*

1.2.2 Calculation of partial charges

In order to set up partial charges for the acyl chains, the *RESP ESP Charge Derive Server* 3.0 [7] was used. Quantum mechanical calculations were performed with Gaussian09 [8]. In analogy to the derivation

of charges for the predefined Glycam residues, the protocol RESP-C2 (HF//6-31G*//HF/6-31G*) was selected.

The charge fit was performed for every acyl chain residue with the respective fatty acid methyl ester. However, the charges for the carbonyl carbon atom, the carbonyl oxygen atom and the methyl moiety were calculated only once for methyl acetate and then set as a constraint for all further systems.

The methyl acetate molecule (structural formula with atom names in Supplementary Fig. S5) was defined to be electrically neutral. Then, a charge constraint of $q = 0$ was posed upon the methyl group connected to the carbonyl carbon atom, and a charge constraint of $q = -0.194\,e$ was set for the entity of ester oxygen atom and the methyl group connected to the ester oxygen.

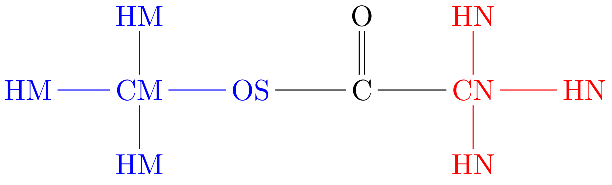

Supplementary Fig. S5. *Structure and atom names of methyl acetate. The methyl group connected to the cabonyl carbon atom (coloured in red) was assigned a charge of $q = 0$, the entity of ester oxygen atom and the second methyl group connected to the ester oxygen (coloured in blue) was assigned $q = -0.194\,e$.*

This approach lead to the implicit assignment of the charge $q = +0.194\,e$ for the carbonyl group (carbonyl carbon and oxygen atom). After an energy optimization of the initial structure, the RESP charge fit lead to the results summarized in Supplementary Table S1.

Supplementary Table S1. *Results for the RESP charge fit of methyl acetate with and without constraints. The Pearson correlation coefficients were $r^2 \approx 0,976$ with and $r^2 \approx 0,981$ without constraints.*

Atom	Derived Charge [e]	
Name	With Constraints	Without Constraints
C	+0.7989	+0.8692
O	−0.6049	−0.6160
OS	−0.4587	−0.4542
CM	+0.0634	+0.0440
HM1	+0.0671	+0.0700
HM2	+0.0671	+0.0700
HM3	+0.0671	+0.0700

In a second step, a RESP fit was calculated for the different acyl chain lengths (`F02`, `F04`, ..., `F22`). Therefore, the structure of the respective fatty acid methyl ester was submitted, and defined to be in total electrically neutral. The partial charges as listed in Supplementary Table S1 were chosen as a constraint. Furthermore, all aliphatic hydrogen atom charges of the alkyl chain were set to zero according to the Glycam force field conventions.

Thereby, it was possible to assign a charge of $q = +0.194\,e$ to our acyl chain residues (carbonyl group + c2 to cn) as required for electrically neutral trehalose acyl esters. Simultaneously, the alkyl chain (c2 to cn) as an entity remained uncharged, but not its individual carbon atoms. The Pearson correlation coefficients determined for the fit of the different fatty acid methyl esters are summarized in Supplementary Table S2.

Supplementary Table S2. *Pearson correlation coefficients for the RESP/ESP charge fit of the different fatty acid methyl esters investigated.*

Fatty Acid Methyl Ester	Pearson Correlation Coefficient r^2	
	With Constraints	Without Constraints
F02	0.976	0.981
F04	0.967	0.973
F06	0.966	0.969
F08	0.958	0.963
F10	0.952	0.957
F12	0.945	0.950
F14	0.939	0.945
F16	0.932	0.939
F18	0.926	0.933
F20	0.920	0.927
F22	0.914	0.921

1.2.3 Generation of prep files and assignment of atom types

The Amber tool `antechamber` was used to convert the PDB files into the prep file format. Then, all prep files were combined into one single file. In order to assign the correct atom types and the partial charges, this file was edited manually. The atom types were assigned following the description in the Glycam parameter file `$AMBERHOME/dat/leap/parm/GLYCAM_06j.dat` and in analogy to the residue `myr` (myristic acid), which is predefined in the Glycam force field (Supplementary Table S3).

Supplementary Table S3. Glycam *atom types used for the acyl chains.*

Atom	Atom type	Remark in parameter file
Carbonyl carbon atom c1	C	sp^2 C carbonyl group
Carbonyl oxgen atom	O	O carbonyl group
Aliphatic carbon atoms c2 – cn	Cg	sp^3 C aliphatic
Hydrogen atoms	Hc	H aliph. bond. to C without electrwd. groups

1.3 Amber prep files

Final versions of the prep files that were created for the glucose unit CGA with free valences at the positions o1 and o6 and for the acyl chains `F02`, `F04`, ..., `F22` are available as Supplementary Data S1 `CGA.tds` and S2 `fs_glycam.tds`, respectively. The file name extension `.tds` instead of `.prep` was used due to Supplementary Data file name restrictions. See the file content for atom names and charges. After sourcing the original Glycam parameters (`source leaprc.GLYCAM_06j-1`), the prep files can be loaded into `leap` with `loadamberprep`. Note that the connectivity between different sugar and/or fatty acid units has to be established manually with the `bond` command.

2 Supplementary Results – Complementary Figures

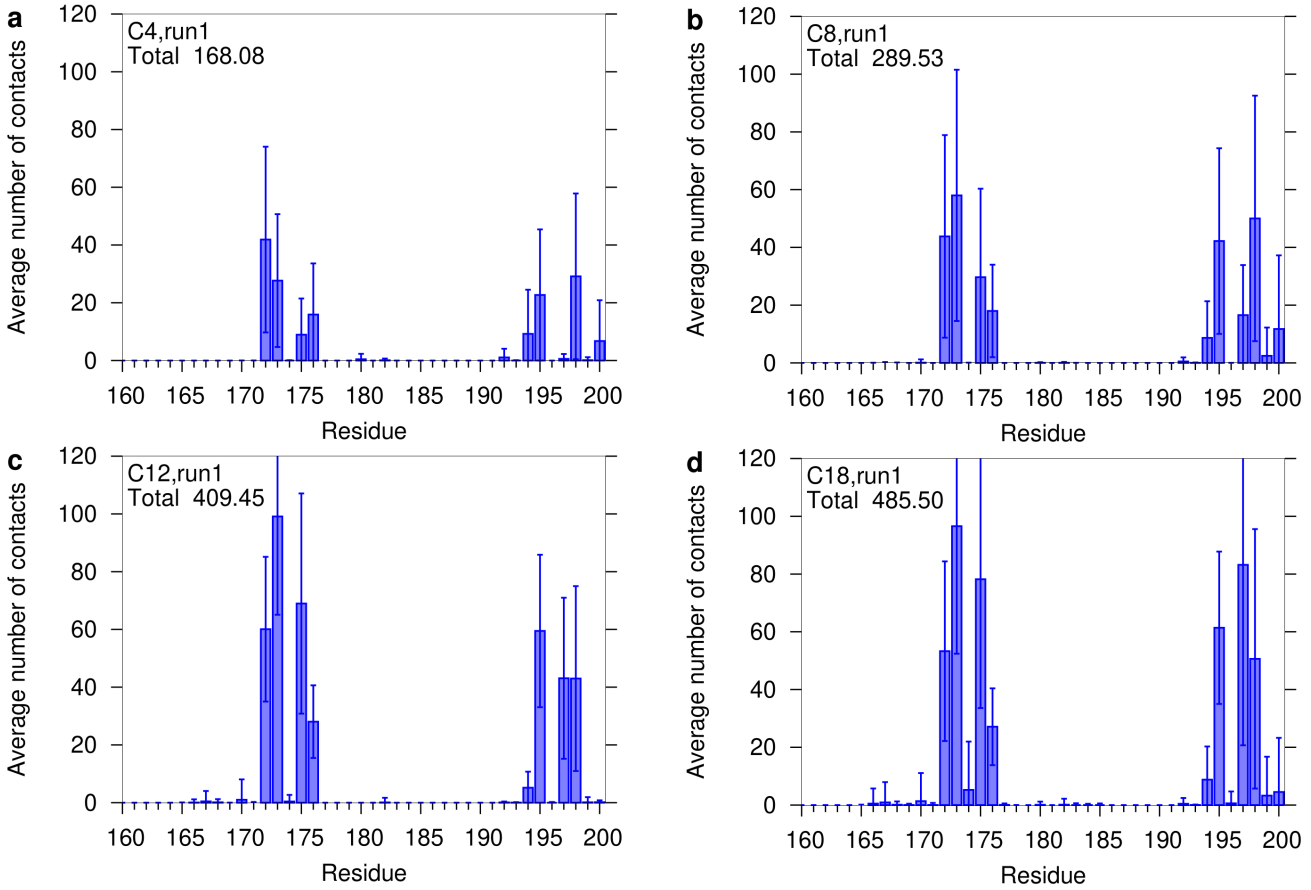

Supplementary Fig. S6. Contacts per Mincle residue for monoesters (run1). *For each Mincle residue, the average number of contacts (± standard deviation) to the acyl chains is plotted. (**a**) C4; (**b**) C8; (**c**) C12; (**d**) C18.*

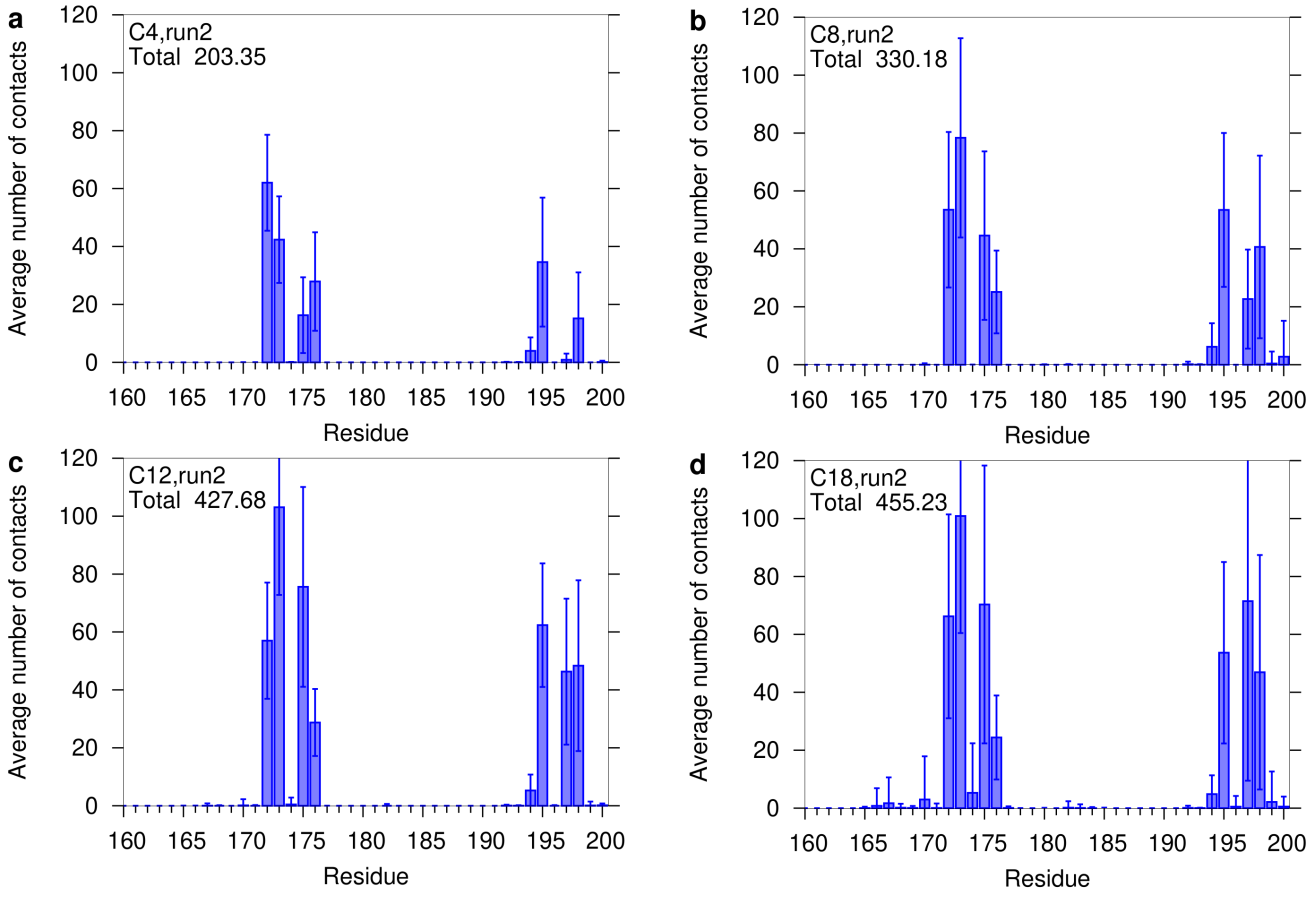

Supplementary Fig. S7. Contacts per Mincle residue for monoesters (run2). *For each Mincle residue, the average number of contacts (± standard deviation) to the acyl chains is plotted. (**a**) C4; (**b**) C8; (**c**) C12; (**d**) C18.*

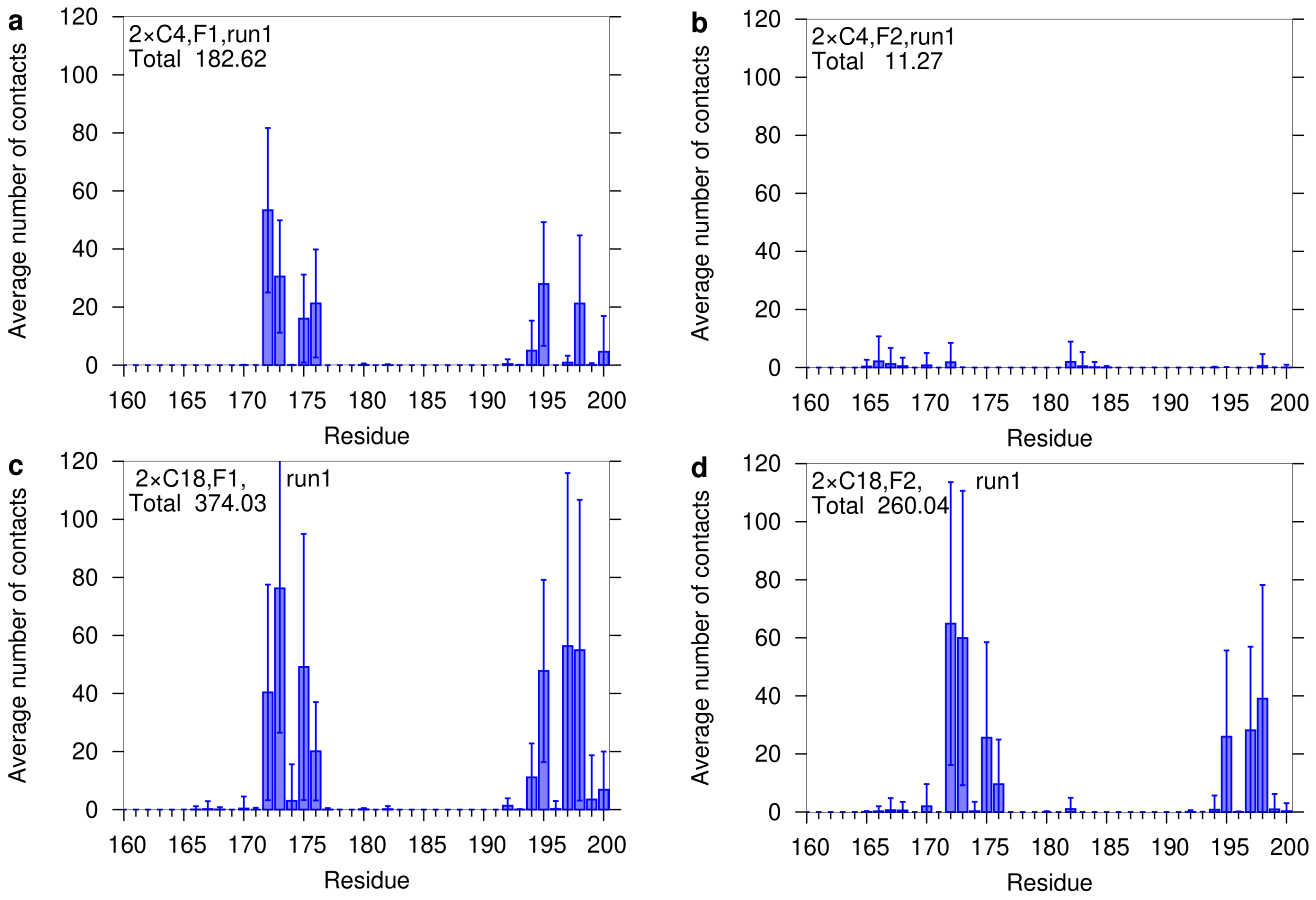

Supplementary Fig. S8. Contacts per Mincle residue for diesters (run1). *For each Mincle residue, the average number of contacts (± standard deviation) to the acyl chains is plotted. (**a**) 2×C4, F1 acyl chain; (**b**) 2×C4, F2 acyl chain; (**c**) 2×C18, F1 acyl chain; (**d**) 2×C18, F2 acyl chain.*

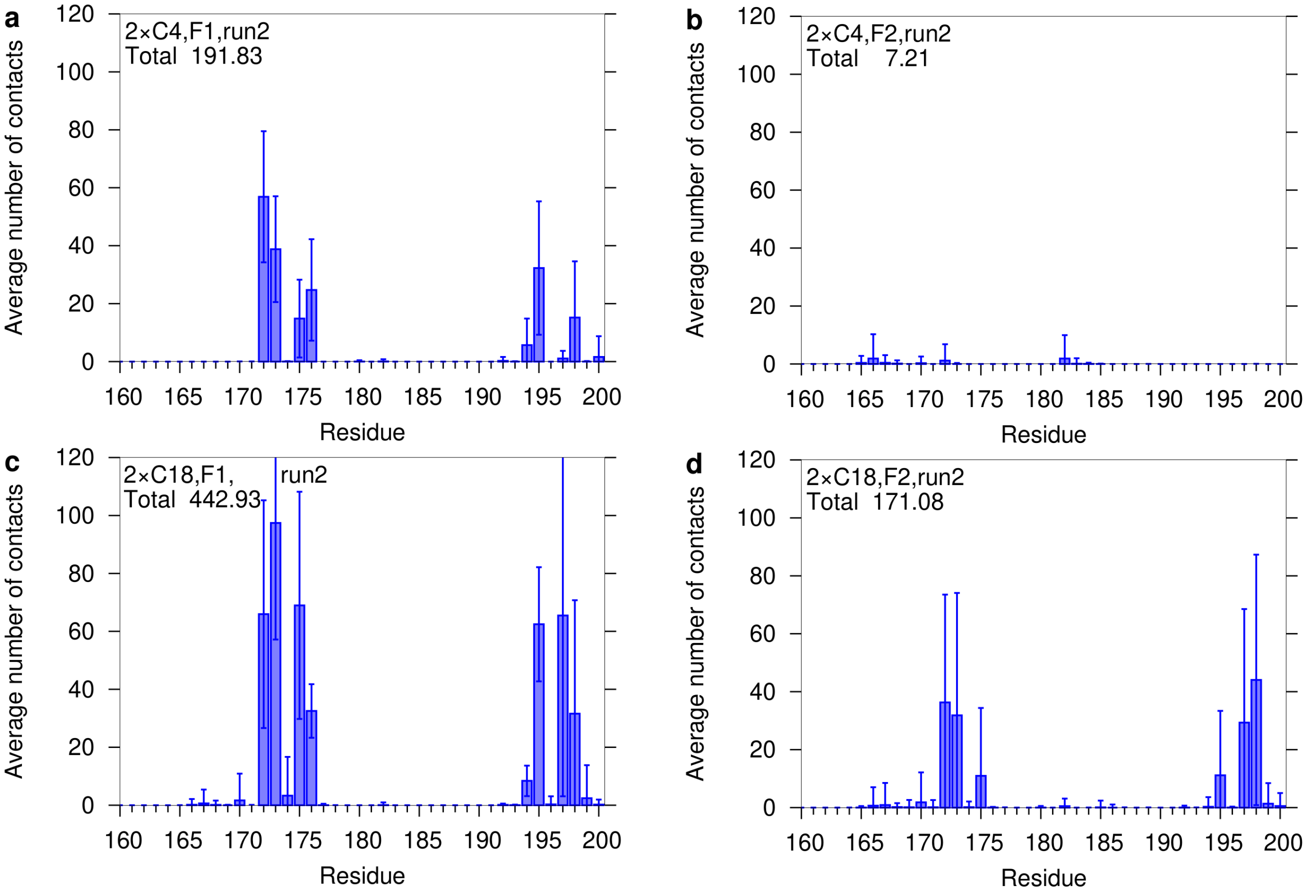

Supplementary Fig. S9. Contacts per Mincle residue for diesters (run2). *For each Mincle residue, the average number of contacts (± standard deviation) to the acyl chains is plotted. (**a**) 2×C4, F1 acyl chain; (**b**) 2×C4, F2 acyl chain; (**c**) 2×C18, F1 acyl chain; (**d**) 2×C18, F2 acyl chain.*

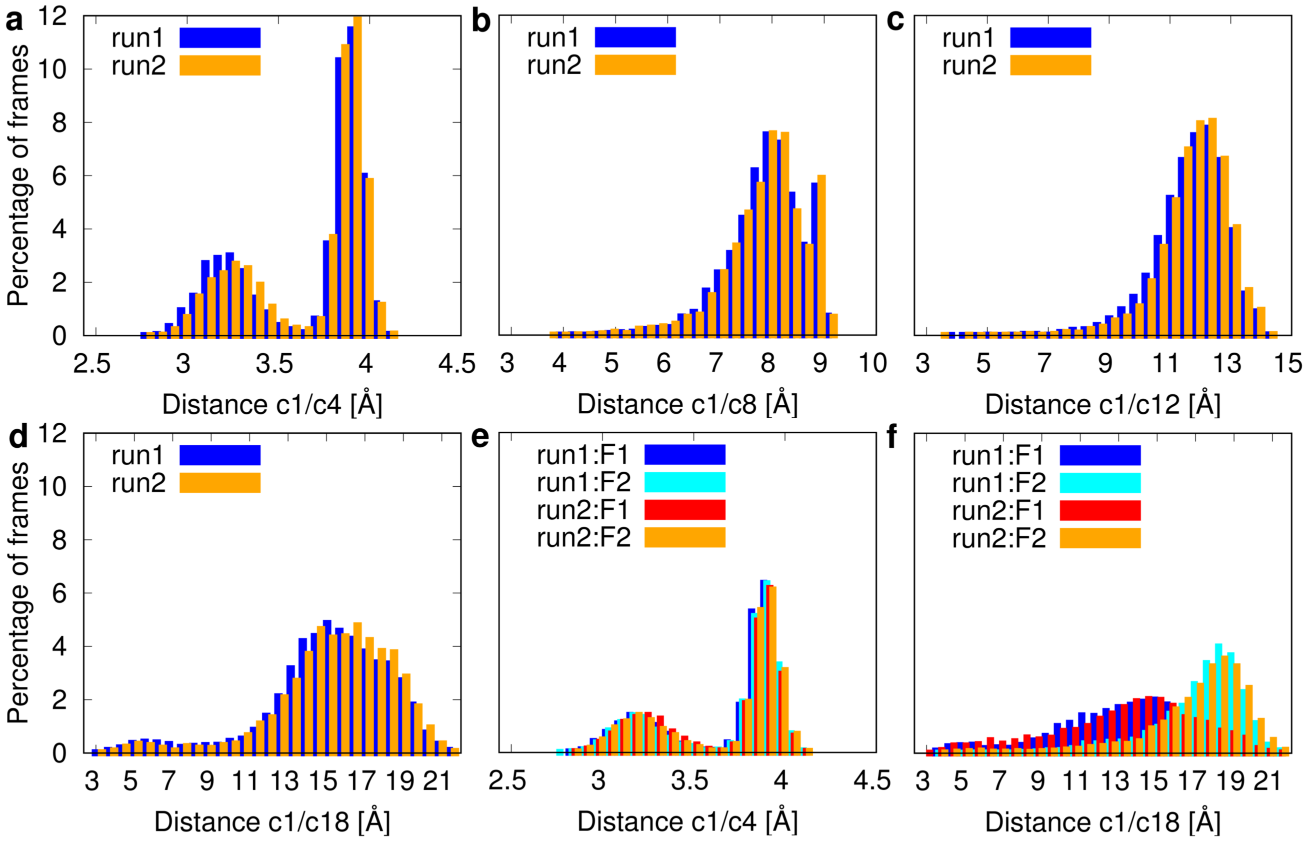

Supplementary Fig. S10. Histograms of the Distances between the First and the Last Carbon Atom of the Fatty Acids. *(**a**) C4; (**b**) C8; (**c**) C12; (**d**) C18; (**e**) 2×C4; (**f**) 2×C18.*

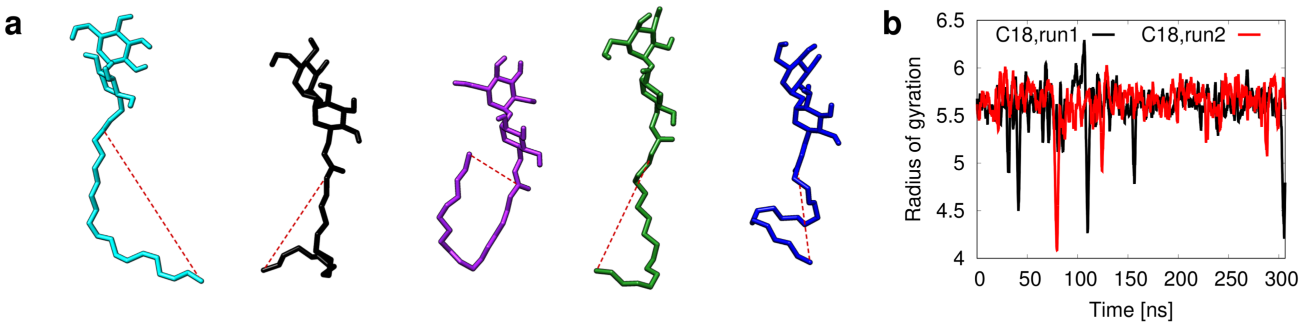

Supplementary Fig. S11. Fatty Acid Flexibility of C18. *(**a**) Five representative cluster structures for C18, run1. The structures are ordered from left to right with decreasing radius of gyration. The distance between the first and the last carbon atom is illustrated as dashed red line. (**b**) Radius of gyration as a function of simulation time for both runs.*

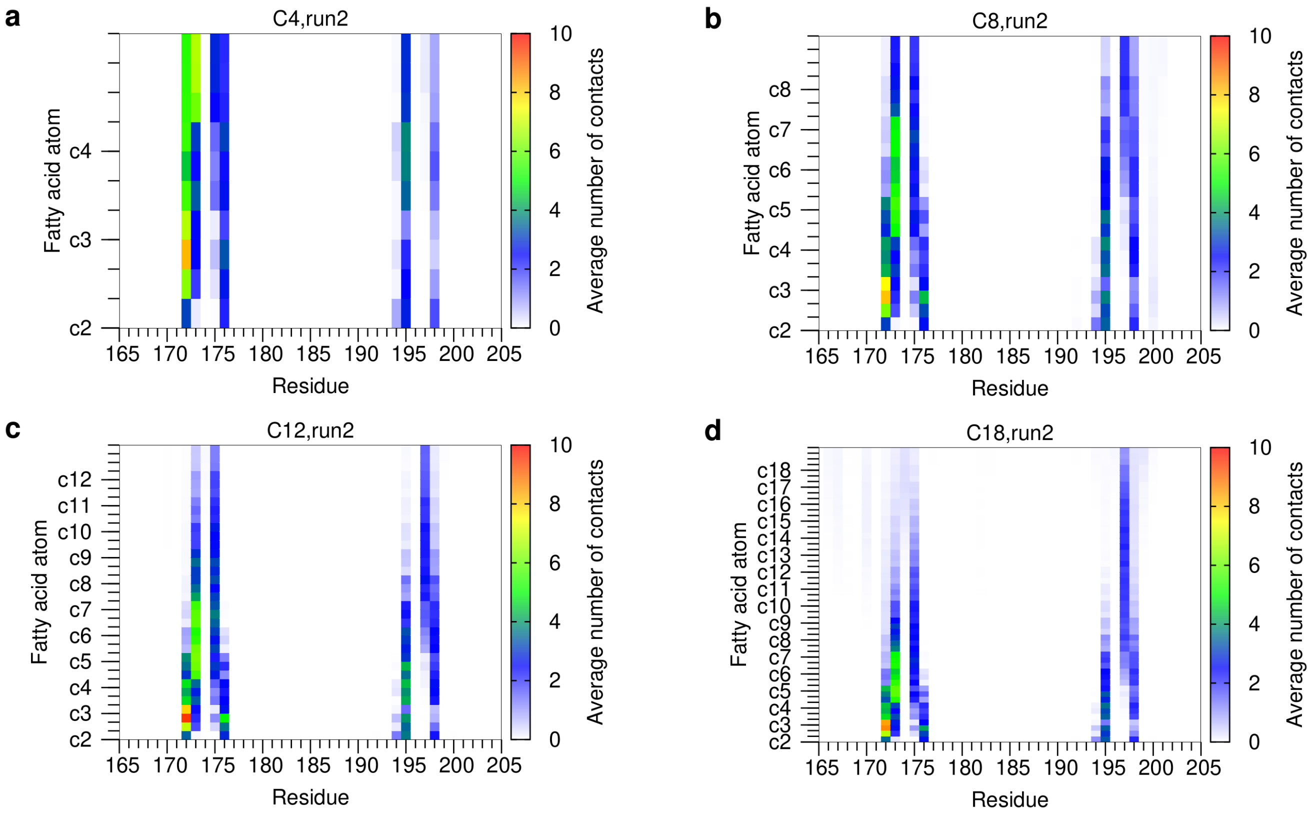

Supplementary Fig. S12. Contacts per Mincle residue and acyl chain atom for monoesters (run2). *(**a**) C4; (**b**) C8; (**c**) C12; (**d**) C18. For each Mincle residue, the contacts with the individual atoms of the acyl sidechains are plotted. For clarity, only the carbon atoms of the acyl chain are labelled on the y-axis, but the bars also display the contacts of the connected hydrogen atoms. For a detailed description of labelling, see Fig. 3 in the main paper. The contact map is colored according to the average number of contacts formed.*

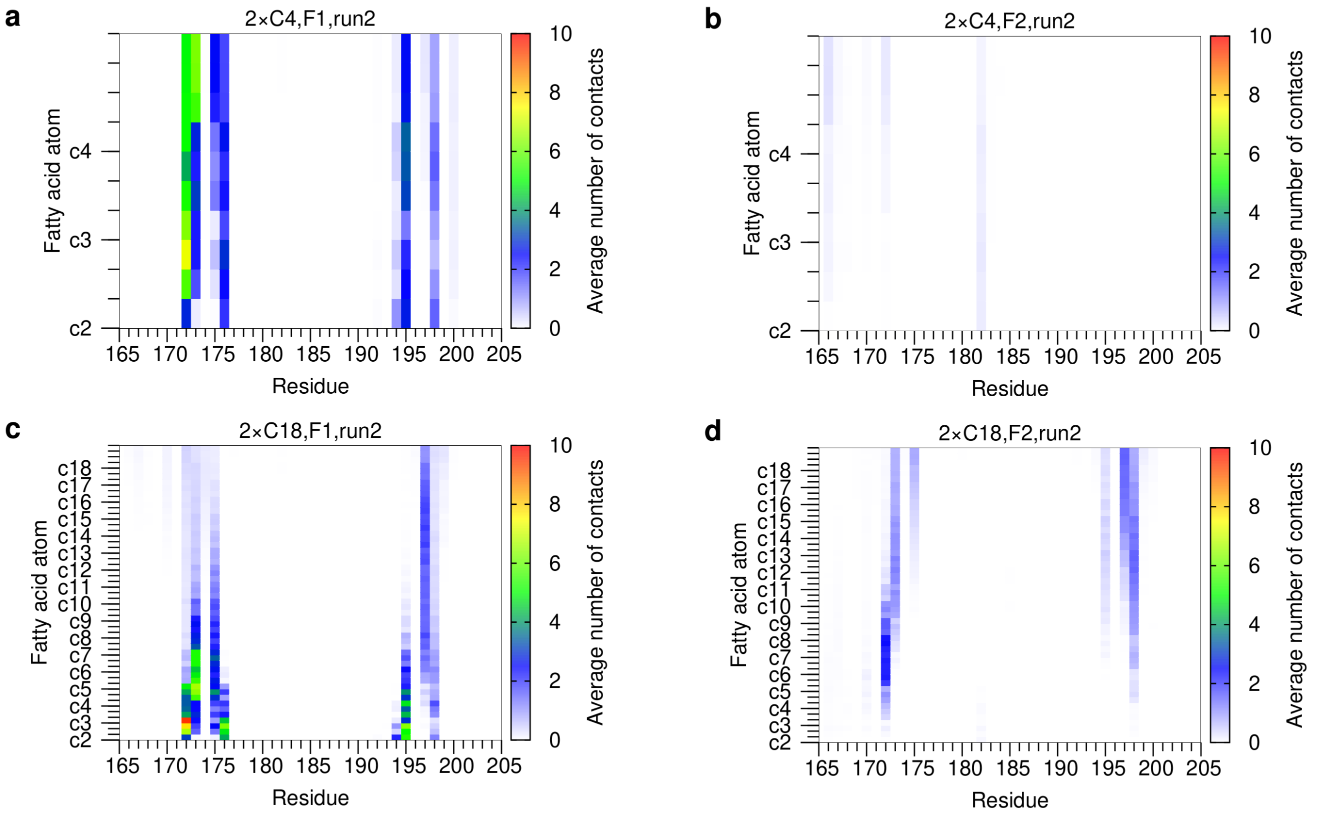

Supplementary Fig. S13. Contacts per Mincle residue and acyl chain atom for diesters (run2). *(**a**) 2×C4, F1 acyl chain; (**b**) 2×C4, F2 acyl chain; (**c**) 2×C18, F1 acyl chain; (**d**) 2×C18, F2 acyl chain. For each Mincle residue, the contacts with the individual atoms of the acyl sidechains are plotted. For clarity, only the carbon atoms of the acyl chain are labelled on the y-axis, but the bars also display the contacts of the connected hydrogen atoms. For a detailed description of labelling, see Fig. 4 in the main paper. The contact map is colored according to the average number of contacts formed.*

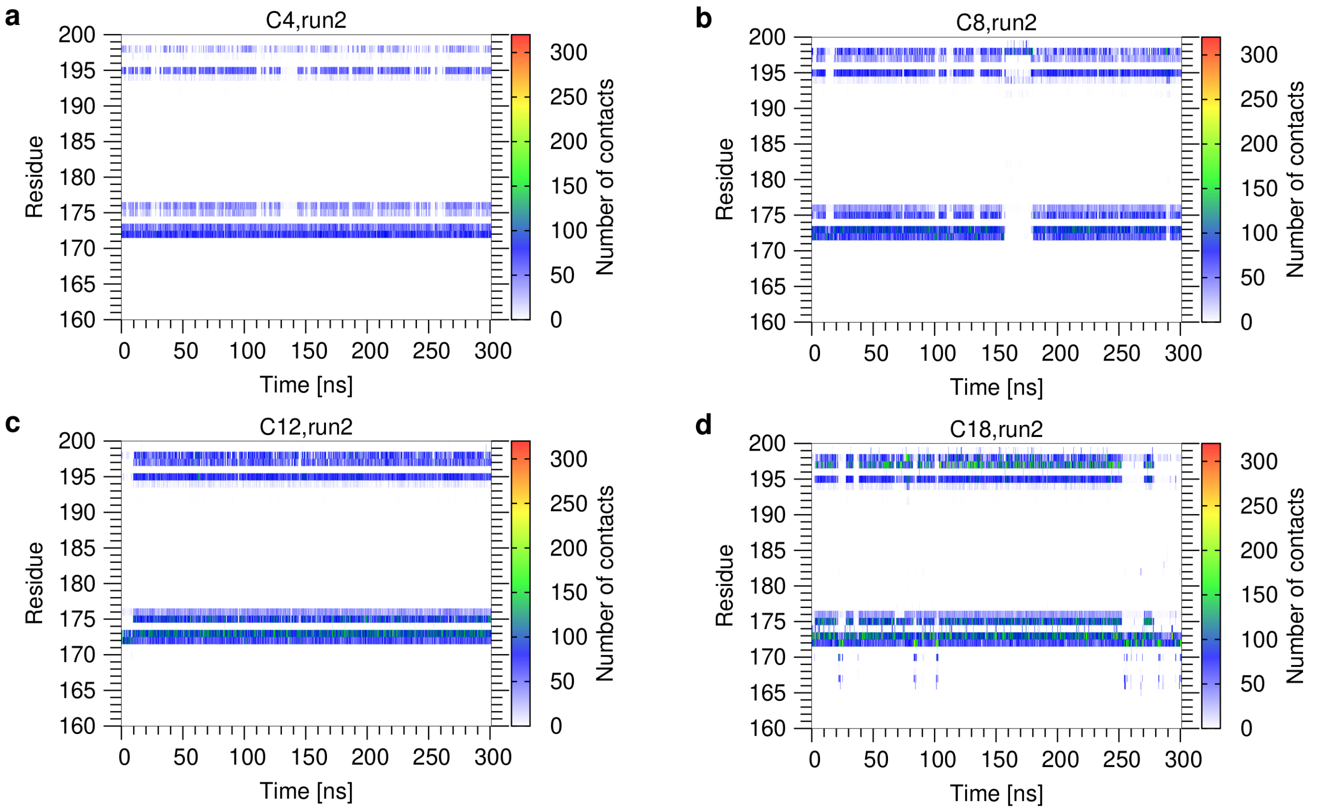

Supplementary Fig. S14. Contacts per Mincle residue as a function of simulation time for monoesters (run2). *(**a**) C4; (**b**) C8; (**c**) C12; (**d**) C18. Contacts were monitored for the individual residues of Mincle (shown on the y-axis) over the simulation time. The contact map is colored according to the number of contacts formed.*

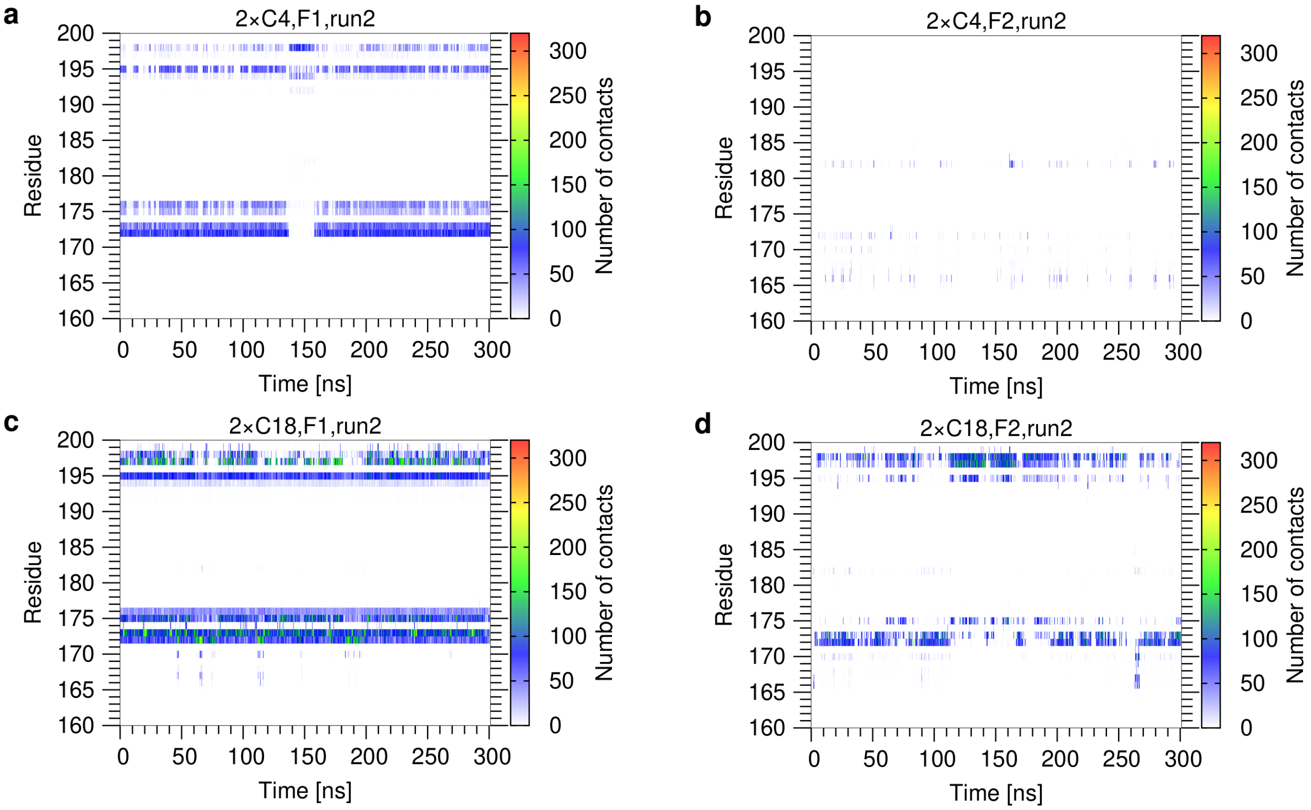

Supplementary Fig. S15. Contacts per Mincle residue as a function of simulation time for diesters (run2). *(**a**) 2×C4, F1 acyl chain; (**b**) 2×C4, F2 acyl chain; (**c**) 2×C18, F1 acyl chain; (**d**) 2×C18, F2 acyl chain. Contacts were monitored for the individual residues of Mincle (shown on the y-axis) over the simulation time. The contact map is colored according to the number of contacts formed. Only residues 160-200 are shown, because almost no contacts were detected with other residues.*

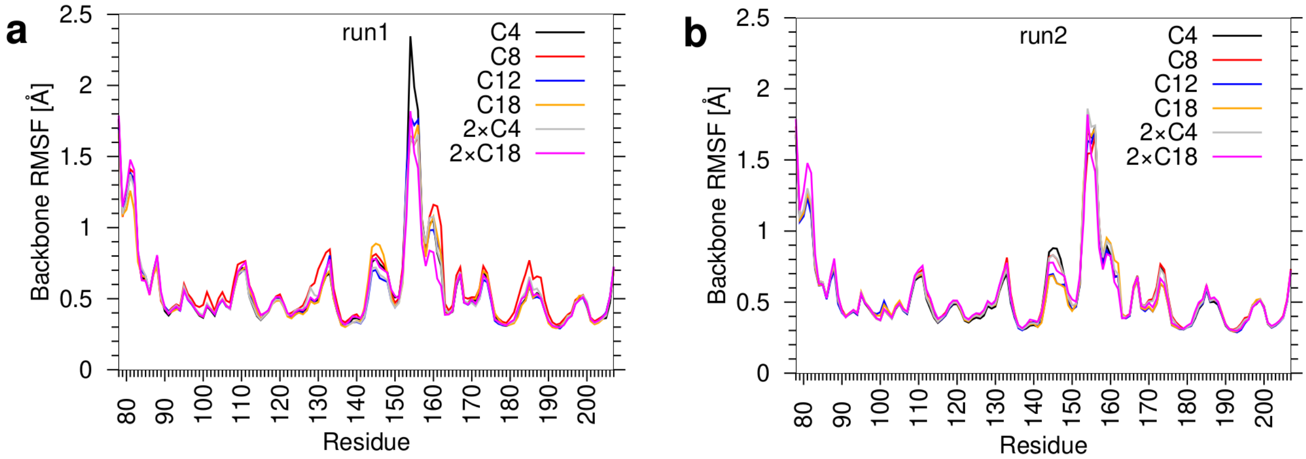

Supplementary Fig. S16. Backbone root-mean-square fluctuations per Mincle residue for the complexes with different glycolipids. *(**a**) run1 (identical to Fig. 8a); (**b**) run2.*

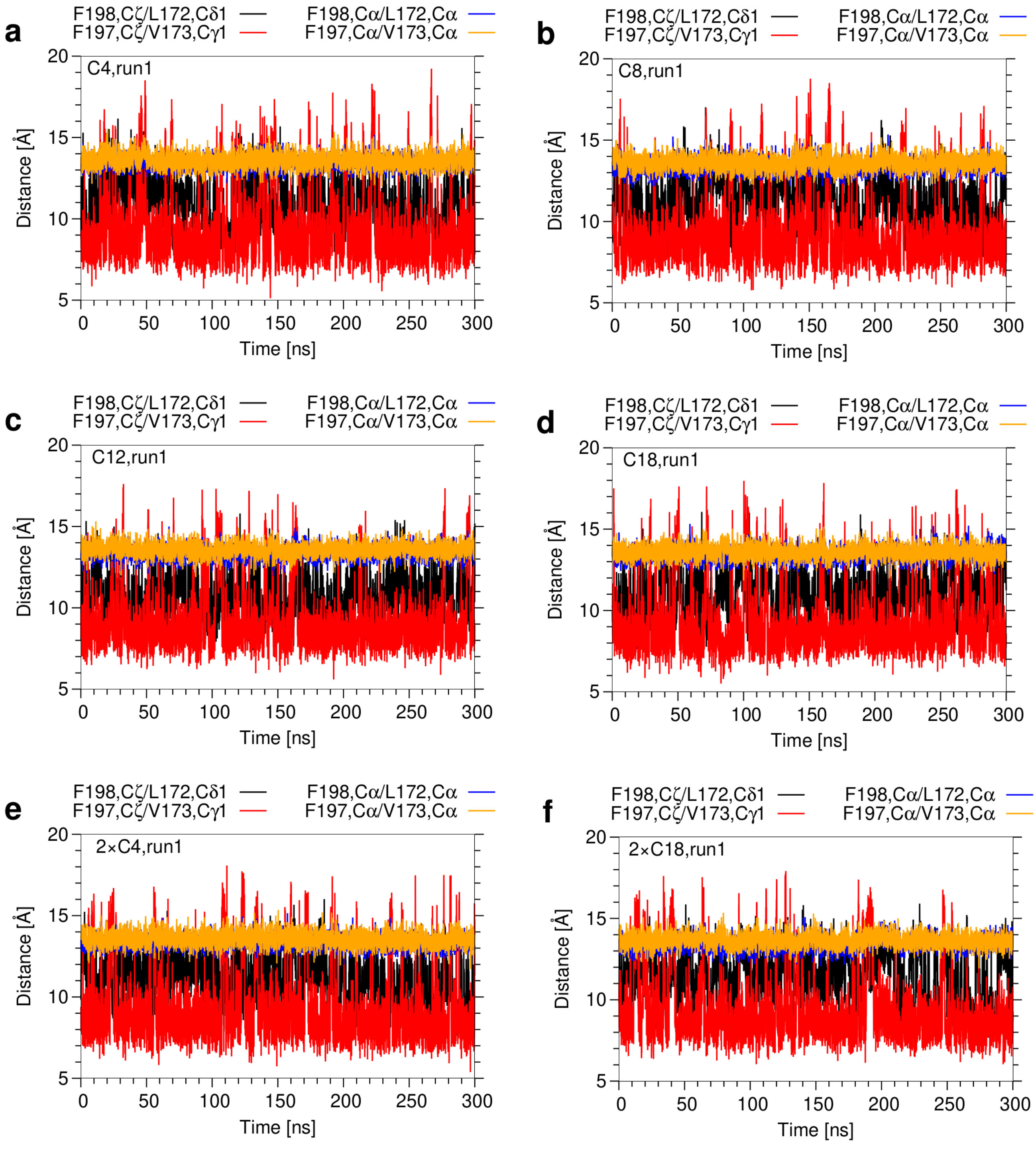

Supplementary Fig. S17. Width of the hydrophobic groove (run1) *for (**a**) C4; (**b**) C8; (**c**) C12; (**d**) C18; (**e**) 2×C4; (**f**) 2×C18 as ligand. Distances between the most distal side chain atoms or between the Cα-atoms were measured for residue pairs lining the hydrophobic groove on opposite sides (i.e. F198-L172 and F197-V173 pairs).*

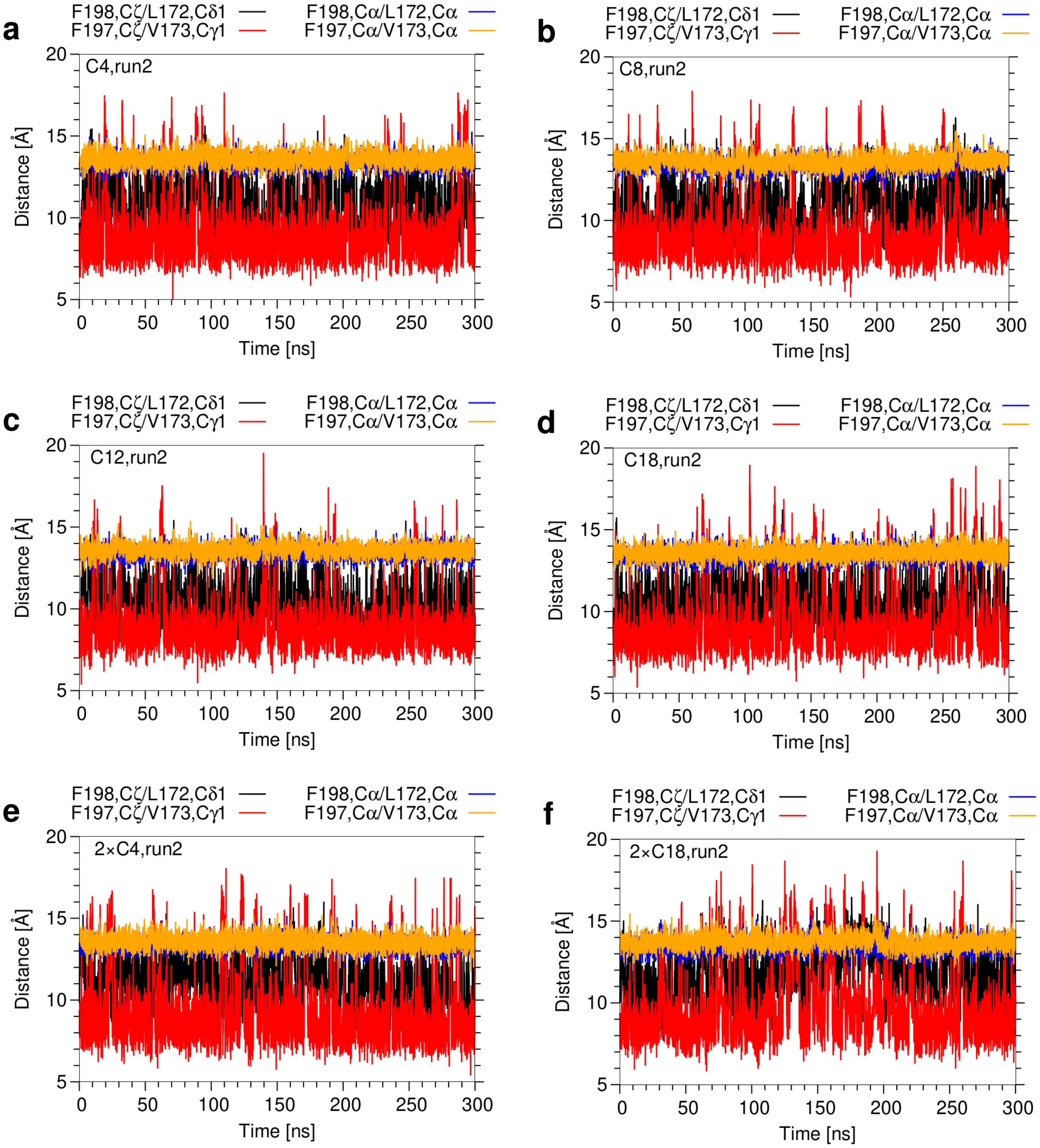

Supplementary Fig. S18. Width of the hydrophobic groove (run2) *for (**a**) C4; (**b**) C8; (**c**) C12; (**d**) C18; (**e**) 2×C4; (**f**) 2×C18 as ligand. Distances between the most distal side chain atoms or between the Cα-atoms were measured for residue pairs lining the hydrophobic groove on opposite sides (i.e. F198-L172 and F197-V173 pairs).*

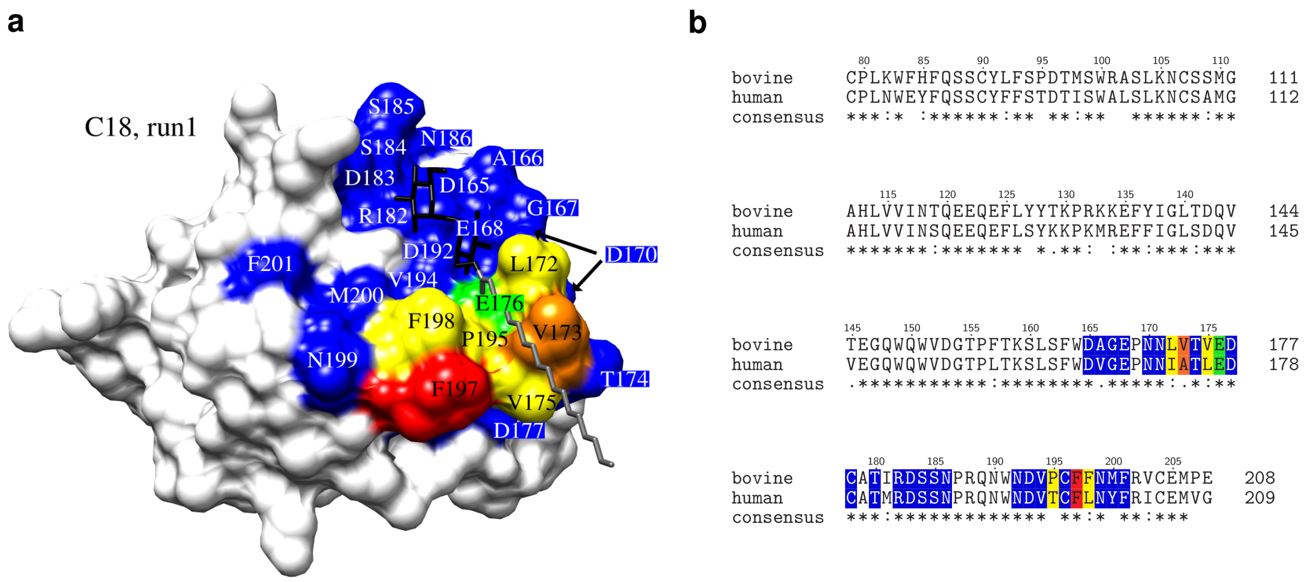

Supplementary Fig. S19. Mincle residues involved in acyl chain interactions. *(**a**) Starting structure of the simulation C18, run1 with Mincle in surface and the ligand C18 in stick representation. The residues are colored according to their average number of contacts with the acyl chain (gray) during the MD trajectory: red > orange > yellow > green > blue. (**b**) Sequence alignment for the carbohydrate recognition domains of bovine Mincle (sequence from PDB* 4ZRV*) and human Mincle (sequence from Uniprot). The alignment was generated using Clustal Omega with default settings. The residues are shaded according to the color code from panel (a).*

References

[1] Kirschner, K. N. *et al.* Glycam06: A generalizable biomolecular force field. Carbohydrates. *J. Comput. Chem.* **29**, 622–655 (2008). URL `http://dx.doi.org/10.1002/jcc.20820`. DOI 10.1002/jcc.20820.

[2] Abel, S., Dupradeau, F.-Y., Raman, E. P., MacKerell, A. D. & Marchi, M. Molecular simulations of dodecyl-β-maltoside micelles in water: Influence of the headgroup conformation and force field parameters. *J. Phys. Chem. B* **115**, 487–499 (2011). URL `http://dx.doi.org/10.1021/jp109545v`. DOI 10.1021/jp109545v.

[3] Chong, T. T., Hashim, R. & Bryce, R. A. Molecular dynamics simulation of monoalkyl glycoside micelles in aqueous solution: Influence of carbohydrate headgroup stereochemistry. *J. Phys. Chem. B* **110**, 4978–4984 (2006). URL `http://dx.doi.org/10.1021/jp056851g`. DOI 10.1021/jp056851g.

[4] Decout, A. *et al.* Rational design of adjuvants targeting the c-type lectin Mincle. *PNAS* **114**, 2675–2680 (2017).

[5] Case, D. A. *et al.* Amber 14. (2014).

[6] Hanwell, M. D. *et al.* Avogadro: an advanced semantic chemical editor, visualization, and analysis platform. *J. Cheminformatics* **4**, 17 (2012). URL `http://dx.doi.org/10.1186/1758-2946-4-17`. DOI 10.1186/1758-2946-4-17.

[7] Vanquelef, E. *et al.* R.E.D. server: a web service for deriving RESP and ESP charges and building force field libraries for new molecules and molecular fragments. *Nucleic Acids Res.* **39**, W511–W517 (2011). URL `http://dx.doi.org/10.1093/nar/gkr288`. DOI 10.1093/nar/gkr288.

[8] Frisch, M. J. *et al.* Gaussian 09, Revision A.1, Gaussian, Inc., Wallingford CT (2009). URL `http://www.gaussian.com/g_prod/g09.htm`.
